# Optic Disc and Optic Cup Segmentation Methodologies for Glaucoma Image Detection: A Survey

**DOI:** 10.1155/2015/180972

**Published:** 2015-11-25

**Authors:** Ahmed Almazroa, Ritambhar Burman, Kaamran Raahemifar, Vasudevan Lakshminarayanan

**Affiliations:** ^1^School of Optometry, Faculty of Science, University of Waterloo, Waterloo, ON, Canada N2L 3G1; ^2^Electronics and Telecommunication Engineering, Jadavpur University, Kolkata 700032, India; ^3^Department of Electrical and Computer Engineering, Ryerson University, Toronto, ON, Canada M5B 2K3

## Abstract

Glaucoma is the second leading cause of loss of vision in the world. Examining the head of optic nerve (cup-to-disc ratio) is very important for diagnosing glaucoma and for patient monitoring after diagnosis. Images of optic disc and optic cup are acquired by fundus camera as well as Optical Coherence Tomography. The optic disc and optic cup segmentation techniques are used to isolate the relevant parts of the retinal image and to calculate the cup-to-disc ratio. The main objective of this paper is to review segmentation methodologies and techniques for the disc and cup boundaries which are utilized to calculate the disc and cup geometrical parameters automatically and accurately to help the professionals in the glaucoma to have a wide view and more details about the optic nerve head structure using retinal fundus images. We provide a brief description of each technique, highlighting its classification and performance metrics. The current and future research directions are summarized and discussed.

## 1. Introduction

Glaucoma is a chronic eye disease in which the optic nerve is gradually damaged. Glaucoma is the second leading cause of blindness after cataract, with approximately 60 million cases reported worldwide in 2010 [1]. It is estimated that by 2020 about 80 million people will suffer from glaucoma [1]. If undiagnosed, glaucoma causes irreversible damage to the optic nerve leading to blindness. Therefore diagnosing glaucoma at early stages is extremely important for an appropriate management of the first-line medical treatment of the disease [2–4].

Accurate diagnosis of glaucoma requires three different sets of examinations: (1) evaluation of the intraocular pressure (IOP) using contact or noncontact tonometry also known as “air puff test” or Goldman tonometry, (2) evaluation of the visual field, and (3) evaluation of the optic nerve head damage [5]. Accurate diagnosis of glaucoma requires more control parameters, that is, gonioscopy and assessment of retinal nerve fibre layer (RNF) [4]. Since both elevated-tension glaucoma and normal-tension glaucoma may or may not increase the IOP, the IOP by itself is not a sufficient screening or diagnosis method [6]. On the other hand, visual field examination requires special equipment which is usually available only in tertiary hospitals, if they have a fundus camera and OCT [6]. In routine practice, patients with POAG can be manifested with inconsistent reports between SD-OCT and SAP. In elderly, higher C/D ratio, larger cup volume, and lower rim area on SD-OCT appear to be associated with detectable VF damage. Moreover, additional worsening in RNFL parameters might reinforce diagnostic consistency between SD-OCT and SAP [7].

Therefore, the optic nerve head examination (cup-to-disc ratio) is the most valuable method for diagnosis glaucoma structurally [8]. The visual field test, on the other hand, diagnoses glaucoma functionally through detecting the damages done to the visual field.

Determining the cup-to-disc ratio is a very expensive and time consuming task currently performed only by professionals. Therefore, automated image detection and assessment of glaucoma will be very useful. There are two different approaches for automatic image detection of the optic nerve head [6]. The first approach is based on the very challenging process of image feature extraction for binary classification of normal and abnormal conditions. The second and more common approach however is based on clinical indicators such as cup-to-disc ratio as well as inferior, superior, nasal, and temporal (ISNT) zones rule in the optic disc area [6].

The optic disc is made of 1.2 million ganglion cell axons passing across the retina and exiting the eye through the scleral canal in order to transit the visual information to brain [8]. Examining the optic disc helps clarify the relationship between the optic nerve cupping and loss of visual field in glaucoma [8]. The optic disc is divided into three different areas: neuroretinal rim, the cup (central area), and sometimes parapapillary atrophy [9]. The cup-to-disc ratio (CDR) is the ratio of the vertical diameter of the cup to the vertical diameter of the disc [10].

Different techniques have been used for optic disc (OD), optic cup (OC), or optic disc with optic cup segmentation. In this paper, we critically review the OD and OC segmentation methodologies that automatically detect OD and OC boundaries. These techniques help professionals with diagnosing and monitoring glaucoma by providing them with clear and accurate information regarding the ONH structure. The uniqueness of this paper is in demonstrating the segmentation methodology by creating a flowchart for each technique. We introduce the algorithms applied to OD and OC segmentation, discuss the pros and cons of each method, and provide suggestions for future research.

The paper is organized in five sections. In Section 2 we describe the materials used for analysis of metrics performance of OD and OC segmentation. In Section 3, the techniques for OD and OC segmentation separately and together are introduced and described.  Section 4 provides a brief discussion. We conclude the paper in Section 5.

## 2. Retinal Image Processing

### 2.1. Fundus Photography

Fundus photography is a complicated process. Fundus camera is equipped with a low power microscope and is designed to capture the image of the posterior pole of the eye as well as the whole retina.

Fundus photography allows three types of examination: (1) color, in which white light is illuminated on the retina to examine it in full color; (2) red-free in which the contrast among vessels and other structures is improved by removing the red color through filtering the imaging light; and (3) angiography in which the contrast of vessels is improved by intravenous injection of a fluorescent dye [11].

### 2.2. Optical Coherence Tomography (OCT)

OCT is an optical signal acquisition method for capturing 3D images with micrometer resolution from within optical scattering media. OCT applies near infrared light.

The long wavelength light has the advantage of penetrating into the scattering medium. OCT is usually used for imaging the retina due to its ability to provide high resolution cross-sectional images [12]. It is also a useful imaging technique in other areas such as dermatology and cardiology [13].

### 2.3. Optic Disc and Optic Cup Segmentation

**Figure 1:**
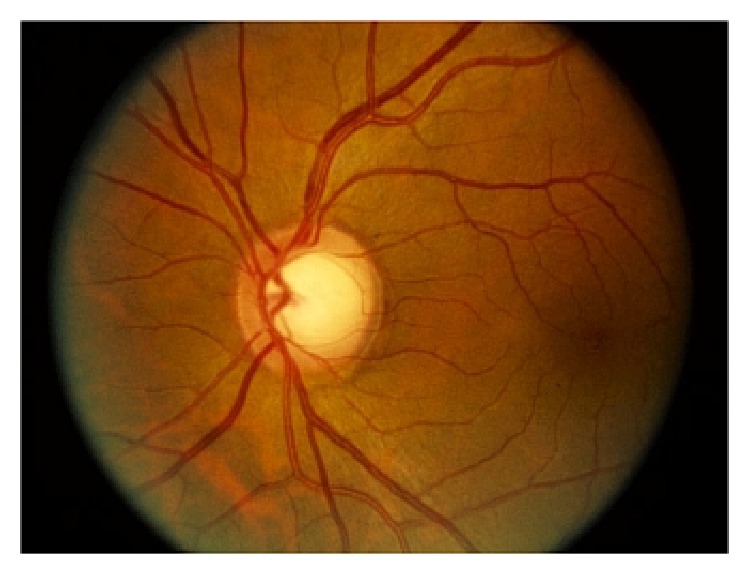
Optic disc in fundus image [14].

Optic disc is one of the most important parts of a retinal fundus image [15] (Figure 1). OD detection is considered a preprocessing component in many methods of automatic image segmentation of retinal structures, a common step in most retinopathy screening procedures [16]. The OD has a vertical oval (elliptical) shape [17] and is divided into two separate zones: the central zone or the cup and the peripheral zone or neuroretinal rim [6].

Changes in the color, shape, or depth of OD are indications of ophthalmic pathologies such as glaucoma [18]; therefore, OD measurements have important diagnostic values [19, 20]. Accurate detection of the central point of OD is important in such measurements. Furthermore, correct segmentation of OD requires accurate detection of the boundary between the retina and the rim [17]. Pathological cases occurring on the OD boundaries, such as papillary atrophy, influence the segmentation accuracy. OC segmentation is further challenged by the density of blood vessels covering parts of the cup and the gradual change in color intensity between the rim and cup. The kinks in the blood vessels sometimes help detecting the cup boundaries and sometimes make it more challenging. Bad image acquisition also affects cup segmentation. Accurate disc and cup segmentation is very important in all pathological cases since errors in disc and cup segmentation may mislead the professionals and hence affect their diagnosis.

### 2.4. Publicly Available Retinal Image Datasets

Most of the retinal optic disc and cup segmentation methodologies presented in this survey are tested on various publicly available datasets, for example, RIGA, DRIVE, STARE, MESSIDOR, and ORIGA. In this section we provide a brief summary of these datasets.

#### 2.4.1. DRIVE Dataset

**Figure 2:**
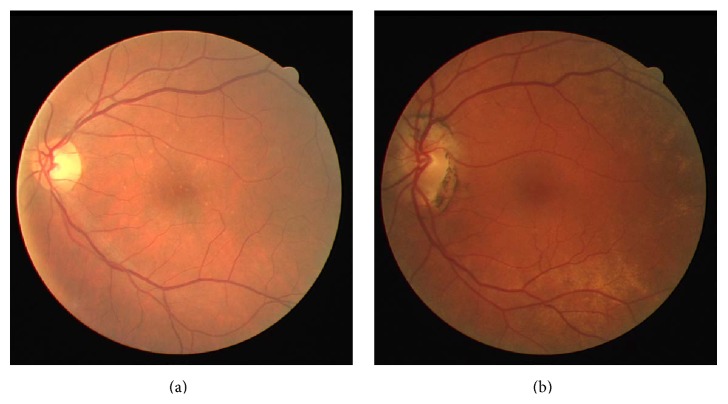
Retinal images from DRIVE: (a) normal image, (b) pathological image.

The Digital Retinal Images for Vessel Extraction (DRIVE) dataset [21] consists of 40 color fundus images. The images were acquired from a diabetic retinopathy research program in Netherlands. Seven images of the dataset have pathology.  Figure 2 shows an example of a normal (Figure 2(a)) and a pathological (Figure 2(b)) image.

A Canon CR5 nonmydriatic 3CCD camera with a 45° field of view was used to obtain the images. The images were divided into two groups, a training set and a test set, with 20 images in each group. Three experts manually segmented the images in order to have reference images for evaluating the techniques by comparing the manually segmented images with those segmented automatically.

#### 2.4.2. STARE Dataset

The Structured Analysis of Retina (STARE) dataset [22] is funded by the US National Institutes of Health. The project has 400 fundus images. Each image is diagnosis. The blood vessels are annotated in 40 images. The ONH is localized in 80 images. A TopCon TRV-50 fundus camera with 35° field of view was used to capture the images.

#### 2.4.3. MESSIDOR Dataset

MESSIDOR [23] contains 1200 images in two sets; the images were captured in three ophthalmological departments by a research program sponsored by the French Ministries of Research and Defense. Two diagnoses are provided by the medical experts for each image, retinopathy grade, and risk of macular edema. A color video 3CCD camera on a Topcon TRC NW6 nonmydriatic retinography with a 45° field of view was used to capture the images. The images are saved in uncompressed TIFF format.

#### 2.4.4. ORIGA Dataset

The Online Retinal Fundus Image Dataset for Glaucoma Analysis and Research (ORIGA) [24] consists of 650 images acquired through Singapore Malay Eye Study (SiMES). Critical signs for glaucoma diagnosis are annotated.

SiMES is conducted by the Singapore Eye Research Institute (SERI). The images were marked by experts based on an algorithm proposed in [25] and are stored in a centralized server. The dataset includes 168 glaucomatous and 482 nonglaucoma images.

#### 2.4.5. DIARETDB0 Dataset

The Standard Diabetic Retinopathy Database Calibration level 0 DIARETDB0 [26] consists of 130 color fundus images, 20 normal and 110 with signs of diabetic retinopathy, acquired from the Kuopio University Hospital in Finland. The images were captured by a digital fundus camera with 50° field of view.

#### 2.4.6. DIARETDB1 Dataset

The diabetic retinopathy database and evaluation protocol DIARETDB1 [27] consists of 89 color fundus images acquired from the Kuopio University Hospital in Finland. The dataset consists of 84 images with diabetic retinopathy and 4 normal images. The images were captured by a digital fundus camera with 50° field of view. Four experts annotated the microaneurysms, hemorrhages, and hard and soft exudates.

### 2.5. Performance Metrics

**Figure 3:**
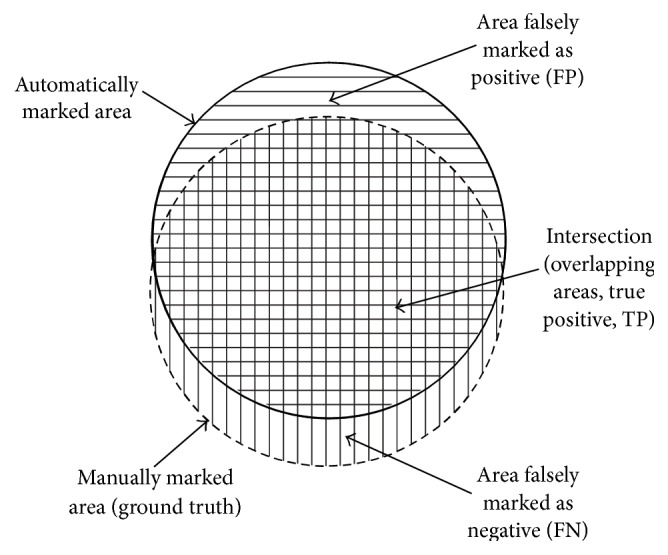
The relation between the ground truth and automatically marked area [9].

The outcome of optic disc segmentation process is pixel based.  Figure 3 shows the three distinctive areas: (1) the true positive area representing the overlapping area between the manually marked (ground truth) and automatically marked (segmented image) areas, (2) the false negative area where a pixel is classified only in the manually marked area, and (3) the false positive area where the pixel is classified only in the automatically segmented area. Sensitivity measures the proportion of the actual positives which are correctly identified. A higher sensitivity value implies a higher validity of results [28].

**Figure 4:**
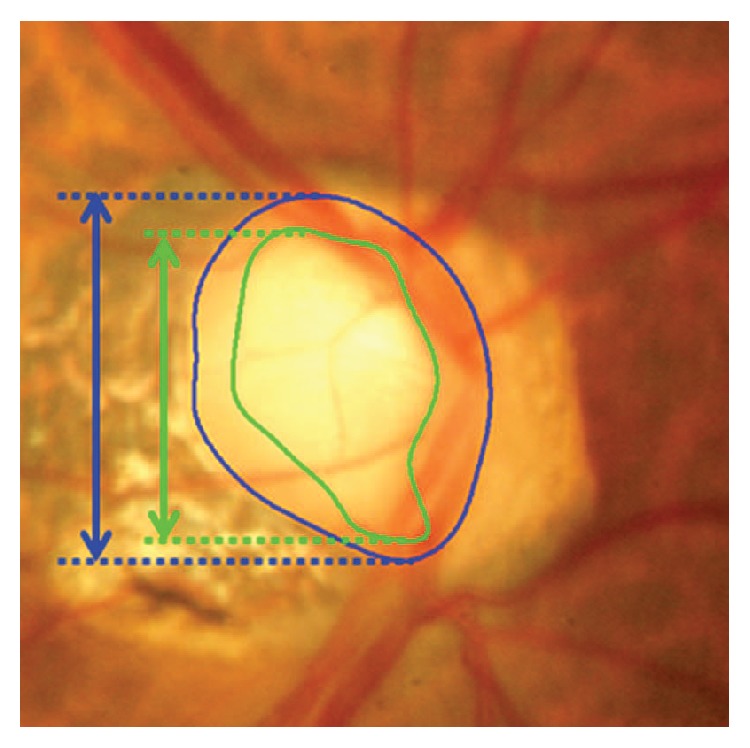
Measurement of cup-to-disc ratio for a tilted disc [44].

On the other hand, there are different measurements used in image classification of the optic disc and optic cup segmentation to determine whether an image is normal or glaucomatous. Cup-to-disc ratio is defined as the ratio of vertical distances between pixels at the highest and lowest vertical position inside the cup and disc region [41] (Figure 4).

Table 1 summarizes the OD and OC segmentation algorithms performance metrics.

**Figure 5:**
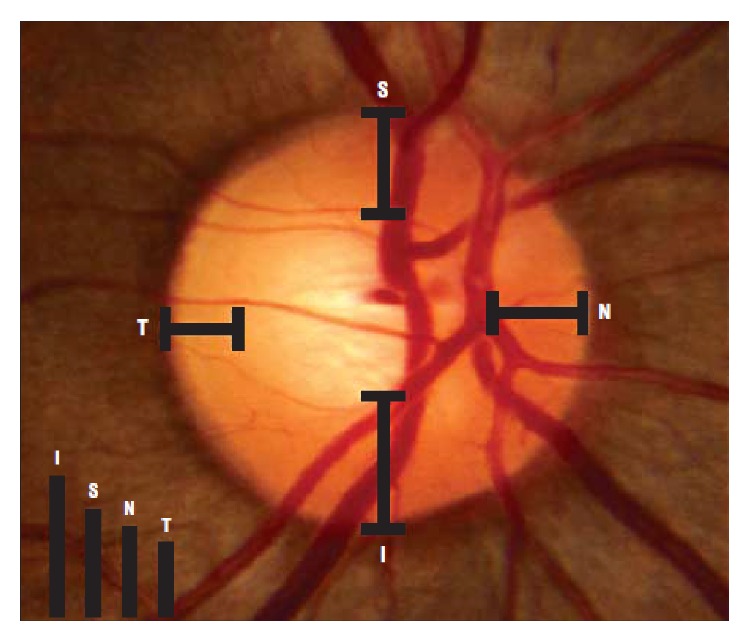
Measurement of the ISNT rule [45].

Various methods are used for image classification which is a clinical assessment of the ISNT rule for the optic nerve [26]. The ISNT rule was considered to be an observer to the gradual decrease or no change in rim width at the following position order: inferior (I) ≥ superior (S) ≥ nasal (N) ≥ temporal (T) (Figure 5).

Sensitivity is the probability of an abnormal class to be identified as abnormal. Specificity is the possibility probability of a normal class to be identified as normal. Accuracy represents the ability or quality of the performance. The positive predictive accuracy represents the precision in detecting the normal and abnormal cases. The true negative represents the number of normal images identified as normal; false negative represents the number of glaucoma images identified as normal; true positive represents the number of glaucoma images identified as glaucoma; and false positive represents the number of normal images identified as glaucoma images [42].

## 3. Segmentation Approaches

In this section technical information will be provided, where there are three main techniques for segmentation, namely, thresholding, edge-based methods, and region-based methods [43]. These techniques have also been applied in the image processing methodologies of the optic disc and optic cup segmentation in this paper.

Here, we consider three segmentation methodologies: (1) optic disc segmentation approaches, (2) optic cup segmentation approaches, and (3) optic disc and optic cup segmentation together. Where most of the papers are concerned with just the optic disc approaches, few are concerned with the optic cup approaches and some are concerned with optic disc and optic cup segmentation together.

### 3.1. Optic Disc Segmentation Approaches

Optic disc extraction or segmentation is performed using segmented reference images called “ground truth” on which the optic disc is accurately marked by ophthalmologists. The OD processing includes two main steps: localization (detecting the center point of OD) and segmentation (detecting the disc boundary) [32]. Different OD detection and segmentation algorithms have already been introduced; however, many of them have a number of limitations [36] such as using images with a clear color variation across OD boundary.

Preprocessing methods are important steps for analyzing an image by enhancing the images and finding the region of interest (ROI). The OD segmentation approaches are summarized in Table 2 and their results are shown in Table 3.

**Figure 6:**
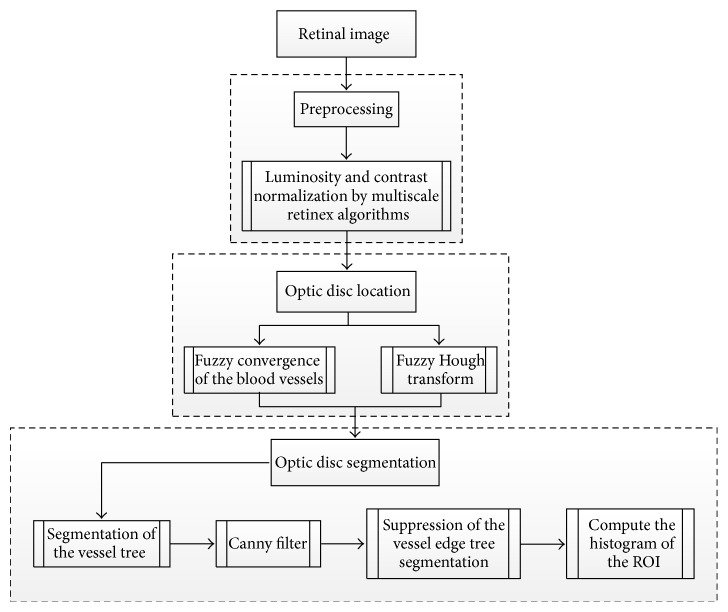
Flowchart for algorithm proposed in [39].

Fraga et al. [39] presented a methodology for the OD segmentation containing different stages (Figure 6). In order to decrease the contrast variability and increase the process reliability, the retinal image was normalized by means of the retinex algorithm [46].

Two different techniques were used to localize the optic disc: (1) analyzing the convergence of the vessels [47] to detect the circular bright shapes and (2) detecting the brightest circular area based on a fuzzy Hough transform [48]. After detecting the OD, the segmentation techniques were conducted using the region of interest specified by a difference of Gaussian filter. The vessel tree boundaries were segmented by Canny filter to compute the edges. The vessels edges from the Canny output were suppressed using the vessel tree segmentation. Finally, the histogram information was included to measure the accuracy of segmentation. The methodology was evaluated on 120 images from the VARIA dataset. The method achieved 100% of OD localization for both fuzzy convergence and Hough transform. Using brute force search, the segmentation success rates were 92.23% and 93.36% for the fuzzy convergence and Hough transform, respectively. The aforementioned OD segmentation approach did not involve pathologic retinal images affecting the OD. This is a limitation which should be addressed in the future work in order to develop a robust methodology.

**Figure 7:**
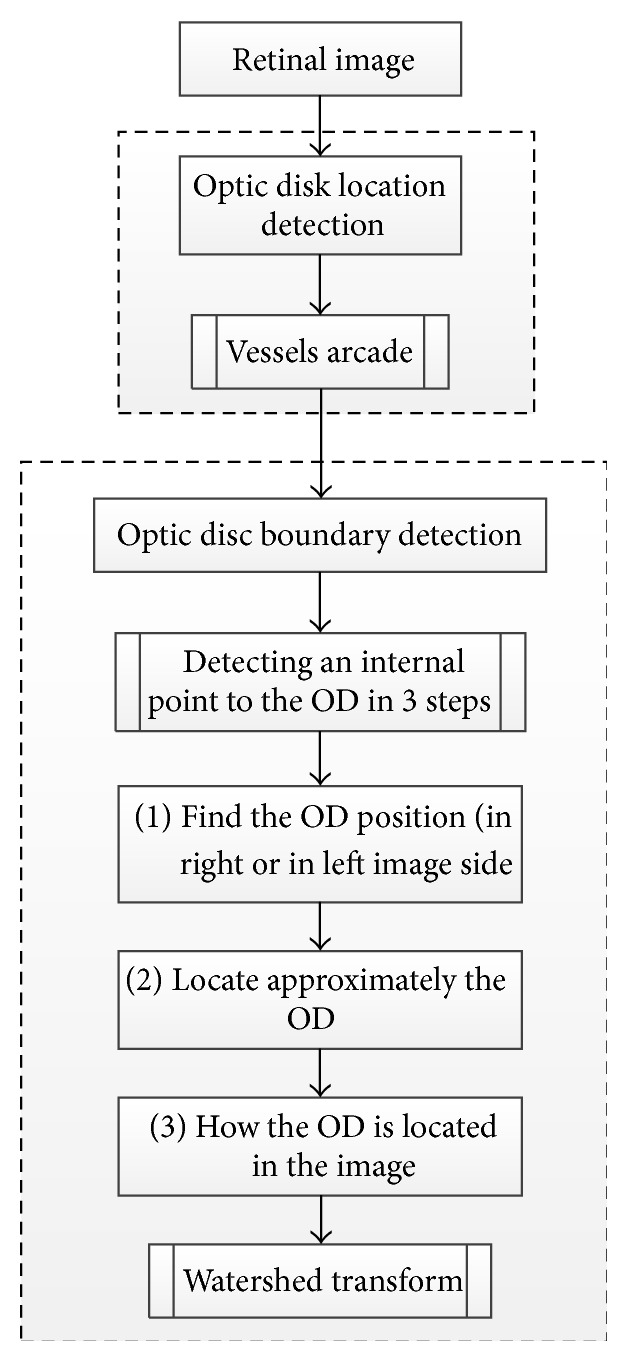
Flowchart for algorithm proposed in [31].

Welfer et al. [31] present a new adaptive method based on a model of the vascular structure using mathematical morphology for the OD automatic segmentation (Figure 7). This methodology has two main stages: (1) detecting the OD location from the information of the main vessels arcade, where the vessels were detected to determine the foreground and background of the green channel image; in this stage, the RMIN operator (which detects the regional minima pixels) was used to identify the background region; (2) detecting the optic disc boundary. In order to detect the OD boundary using the watershed transform, based on the previously detected vascular tree an internal point to the optic disc and other points in vicinity of the internal point were identified using the following three steps: (1) using a specific algorithm to find the OD position and to determine whether it is on the right or left side of the image (morphological skeleton and pruning cycle are used in this step), (2) locating the optic disc by removing the less important vessels from the pruned image, (3) describing the shape of the optic disc. The methods were tested on 40 images obtained from DRIVE dataset and 89 images from DIARETDB1 dataset. The success rate in optic disc localization was 100% and 97.75% for the DRIVE and DIARETDB1 datasets, respectively. Future works should consider detecting other important retina structures, such as fovea, based on the proposed method.

**Figure 8:**
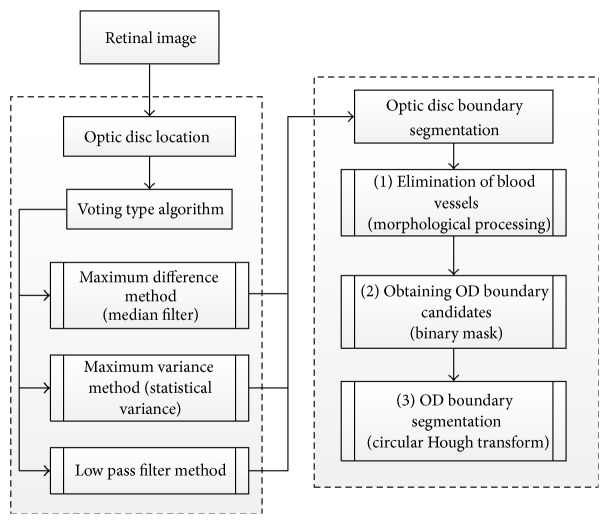
Flowchart for algorithm proposed in [32].

**Figure 9:**
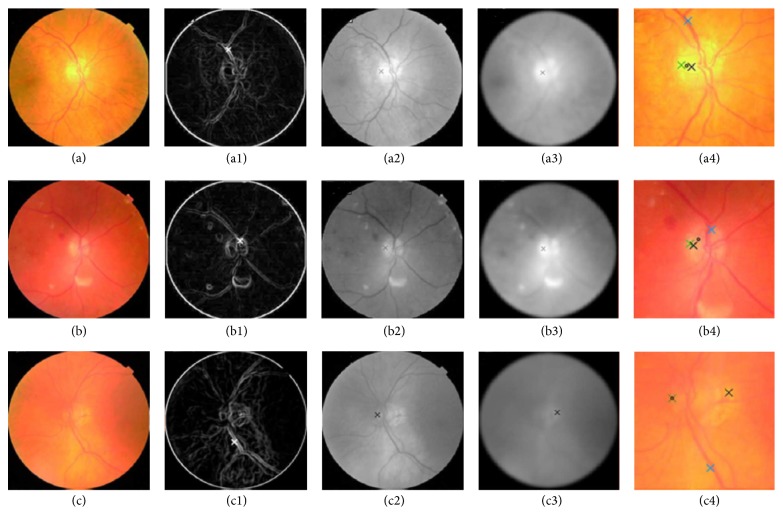
ODP determination. ((a), (b), and (c)) Original images. ((a1), (b1), and (c1)) OD pixels provided by the maximum difference method. ((a2), (b2), and (c2)) OD pixels provided by the maximum variance method. ((a3), (b3), and (c3)) OD pixels provided by the low-pass filter method. ((a4), (b4), and (c4)) Final ODP determination.

**Figure 10:**
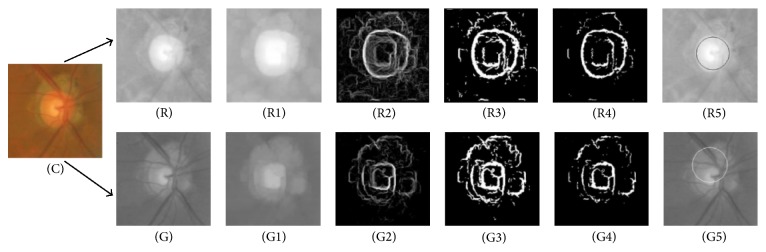
The calculation process of the circular OD boundary approximation. (R) Red channel. (G) Green channel. ((R1) and (G1)) Vessel elimination. ((R2) and (G2)) Gradient magnitude image. ((R3) and (G3)) Binary image. ((R4) and (G4)) Cleaner version of the binary image. ((R5) and (G5)) Circular OD boundary approximation.

Aquino et al. [32] proposed a new algorithm for OD segmentation (Figure 8), where the localization methodology obtains a pixel from the OD called optic disc pixel. The methodology contains three different detection methods (Figure 9). Each method has its own OD candidate pixel, and the final pixel is chosen by a voting procedure. The green channel has been selected since it provides the best contrast. Two of the three detection methods are called maximum difference method and maximum variance method. In general the maximum variation occurs between the bright region (OD) and the dark region (blood vessels in the disc). Therefore, the maximum variation was used to select the OD pixel of those two methods. In addition, statistical variance for every pixel was calculated in the maximum variance method and the bright pixels were obtained by blue channel thresholding via Otsu method [49]. The last method was low pass filter method, where the OD pixel was the maximum gray level pixel in the filtered image. Finally, the maximum variance method has been chosen as the final OD pixel according to the voting procedure. On the other hand, the OD segmentation methodology was applied on two components “red and green” and the better segmentation was selected (Figure 10). The procedure was based on removing the blood vessels by employing a special morphological processing and then applying edge detection and morphological techniques to obtain a binary mask of the OD boundary candidates. Finally, the circular approximation of the OD was computed using a circular Hough transform. The methodology was evaluated using the publicly available MESSIDOR dataset. The localization was successful in 99% and 86% of the segmentation. The current research is concentrated on improving the algorithm for executing a controlled elliptical deformation of the obtained circumference.

**Figure 11:**
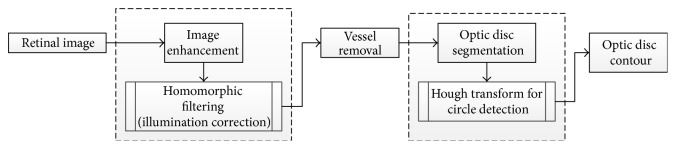
Flowchart for algorithm proposed in [33].

Tjandrasa and colleagues [33] applied the Hough transform as an initial level set for the active contours for optic disc segmentation. The algorithm procedure is shown in Figure 11. The OD segmentation steps start by converting the image to a grayscale image and then implementing the image preprocessing (image enhancement). Therefore, homomorphic filtering is applied to reduce the effect of uneven illumination. Homomorphic filtering has two stages: (1) applying a Gaussian low pass filter, (2) obtaining the filtered edge by performing dilation. The blood vessels are removed in the next step to facilitate the segmentation process. The threshold is applied to detect the low pixel values in the image and followed by applying the median filter to blur the blood vessels. The next step in OD segmentation is detecting a circle which matched the location of OD by performing a Hough transform. Subsequent to this, an active contour model is used to obtain the OD boundaries that are as close to the original OD boundaries as possible. The active contour model is applied with a special processing termed Selective Binary and Gaussian Filtering Regularized Level Set (SBGFRLS) [50]. The algorithm achieved 75.56% of accuracy using 30 images from DRIVE dataset. Further work can be done to segment the cup disc in order to classify the images into normal and glaucomatous.

**Figure 12:**
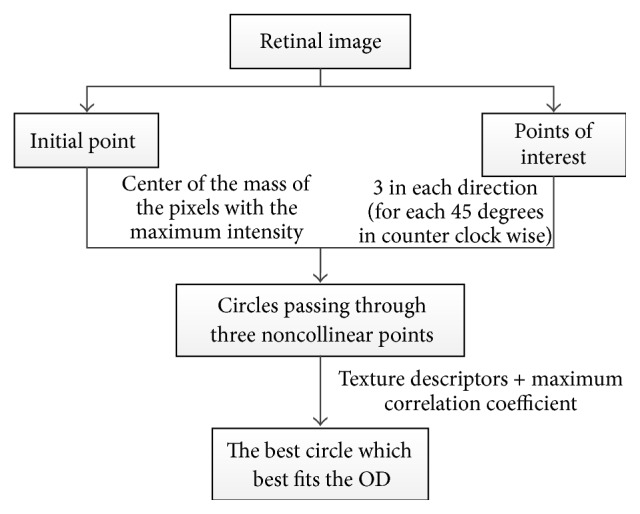
Flowchart for algorithm proposed in [29].

Lupaşcu and colleagues [29] presented an alternative technique (Figure 12) to detect the best circle that matches the OD boundary.

The technique uses a regression based method and texture descriptors to identify the circle which fits the OD boundary. The variation in the intensity of pixels describes the appearance of the OD, and therefore it was used in this algorithm for detecting OD. Since the color fundus images have a dark background the background pixels were not considered. A mask image is computed with zero values for background pixels and one for the foreground pixels. The maximum intensity pixels within the green component provide the highest contrast and therefore were selected. The initial point was established based on the center of the mass of the region, where eight directions, 45° apart from each other, were considered. The directions were obtained by moving counter clockwise in steps of 45°. Each direction was based on the rapid variation of intensity. Three points were considered for each direction; thus in total there were 24 points. The Euclidean distances (the distances between the initial point and each of the 24 points of interest) were computed and their mean value was calculated. The circles were created using three noncollinear points. Hundreds of circles were obtained; however, based on their specific properties, less than twenty circles were selected as the better ones and the rest were removed. Using bilinear filtering the selected circles were mapped into polar coordinate space. The next step was to find the maximum derivatives in *y* direction by applying the linear least squares fitting technique. The correlation coefficient was computed to measure the quality of the fitting. The circle with the maximum correlation coefficient was chosen as the best circle matching the OD. The algorithm was tested on 40 images. An ophthalmologist manually marked the ground truth of OD boundary using standard software to select some pixels on the OD boundary. The success rate was 95% for OD localization and 70% for OD contour (circle) identification. This method causes false detection of OD in low quality images; therefore, further study is needed to improve the algorithm by refining the selection of the initial points.

**Figure 13:**
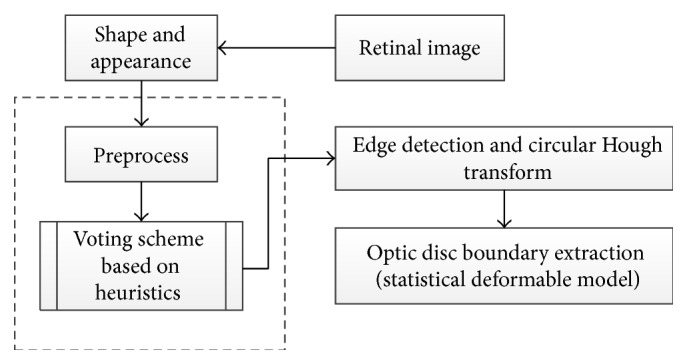
Flowchart for algorithm proposed in [34].

Yin et al. [34] have recently proposed a novel technique that consists of edge detection, circular Hough transform, and a statistical deformable model to determine OD (Figure 13). The Point Distribution Model was utilized to model the shape of the disc using a series of landmarks. A preprocessing step was performed to analyze the image and reduce the effect of blood vessels. The optimal channel was also selected by applying a voting scheme based on heuristics.

**Figure 14:**
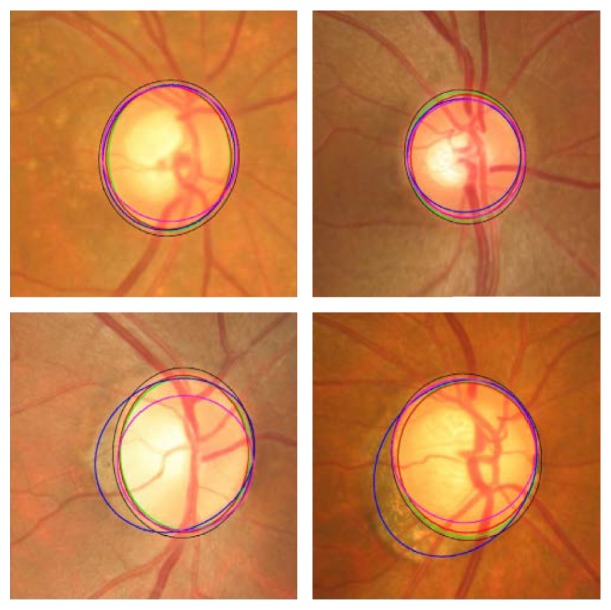
Optic disc segmentation using the proposed method (red), level set method (blue), FCM method (black), CHT method (cyan), and ground truth (green).

Subsequently the OD was approximated by a circle using circular Hough transform to determine the optic disc center and diameter. Ultimately, the statistical deformable model was applied to fine tune the disc boundary according to the image texture. The direct least squared ellipse fitting method was executed to smooth the OD boundary (Figure 14). The ORIGA dataset was used to test the algorithm. The average error in the overlapping area was 11.3% and the average absolute area error was 10.8%.

**Figure 15:**
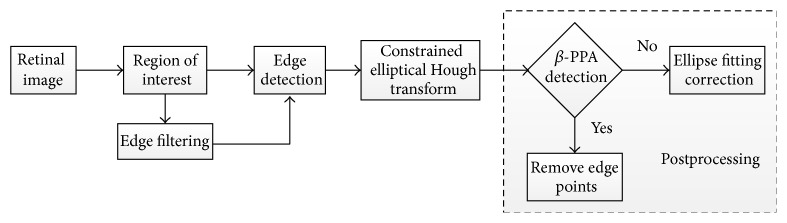
Flowchart for algorithm proposed in [35].

**Figure 16:**
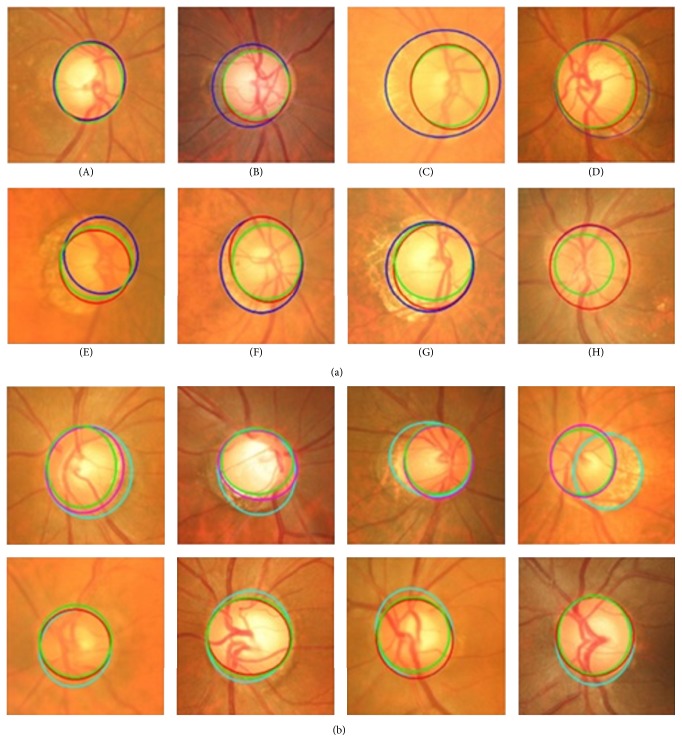
(a) The results (blue: without EF, red: with EF, and green: ground truth). (b) The results (cyan: before *β*-PPA detection, magenta: after *β*-PPA detection, red: with ellipse correction, and green: ground truth).

Cheng et al. [35] proposed an OD segmentation method based on peripapillary atrophy (PPA) elimination. The algorithm included three parts: edge filtering, constraint elliptical Hough transform, and *β*-PPA detection (Figure 15). Extracting the region of interest and detecting the edges of OD were the initial steps in this algorithm. In the aforementioned steps a low pass filter was applied to remove the noise, and then the first derivative from each row of the region of interest (ROI) was computed. The first PPA elimination was edge filtering (EF). There are two types of PPA: *α* and *β*. *α*-PPA is pigmentary and includes a structural irregularity of retinal pigment epithelial cells (darker than OD), while *β*-PPA is a complete loss of the retinal pigment epithelial cells (similar color to OD). The *α*-PPA was simply detected by comparing the ROI with the threshold, that is, the mean intensity in the ROI, followed by a morphological closing processing. Due to the elliptical shape of PPA together with OD, a second elimination of PPA was conducted by a constrained elliptical Hough transform. Finally, the third PPA elimination was conducted by *β*- PPA detection. *β*-PPA is much more difficult than *α*-PPA due to the similarity of its color with that of OD. To avoid false segmentation between the PPA and the OD, a ring area was determined from the detected disc boundary and was divided into quarters. Inspired by the texture within *β*-PPA compared with OD, the local maximums and minimums were extracted within the ring and were named as feature points. *β*-PPA was considered present in a quadrant when the number of feature points in a quadrant exceeded the threshold. The threshold level was obtained by comparing the cases with and without *β*-PPA. Then the edge points along the detected disc boundary were removed from the quadrant. Finally, the constrained elliptical Hough transform was reapplied to obtain the new disc boundary (Figure 16). The ORIGA dataset with 200 images with PPA was used to evaluate the algorithm. Results showed an average overlapping error of 10%, an average absolute area error of 7.4%, and an average accuracy vertical disc diameter error of 4.9%. In the future studies, the method should be reapplied to segment OC for diagnosis of glaucoma.

**Figure 17:**
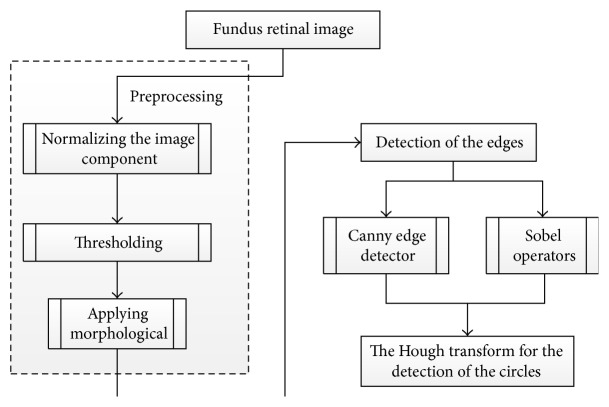
Flowchart for algorithm proposed in [30].

Zhu and Rangayyan [30] proposed an automated segmentation method based on Hough transform to detect the center as well as the radius of a circle that approximates the boundary of OD (Figure 17). Gonzalez and Woods in [51] and Canny in [52] also used this method to detect the edge of OD. To calculate reference intensity for circle selection, a preprocessing step was conducted by normalizing the color image components and converting them to luminance components and then thresholding the effective region of the image. Finally, morphological erosion was used to remove the artifacts from the DRIVE dataset which was used to test this algorithm. A median filter was applied to remove outliers from the image. The components of horizontal and vertical gradient of the Sobel operator were obtained by convolving the preprocessed image with specified operators. The binary edge map was obtained by a threshold applied to the gradient magnitude image. On the other hand, Canny operator was applied to detect the edges based on three criteria: multidirectional derivatives, multiscale analysis, and optimization procedures. After edge detection, Hough transform was applied to detect the center and radius of the circle. The algorithm was tested on two datasets: DRIVE and STARE. The algorithm achieved 92.5% (DRIVE) and 40.24% (STARE) success rates for Sobel method, and 80% (DRIVE) and 21.95% (STARE) success rates for Canny method. The algorithm needs to be improved by applying additional characteristics of OD.

**Figure 18:**
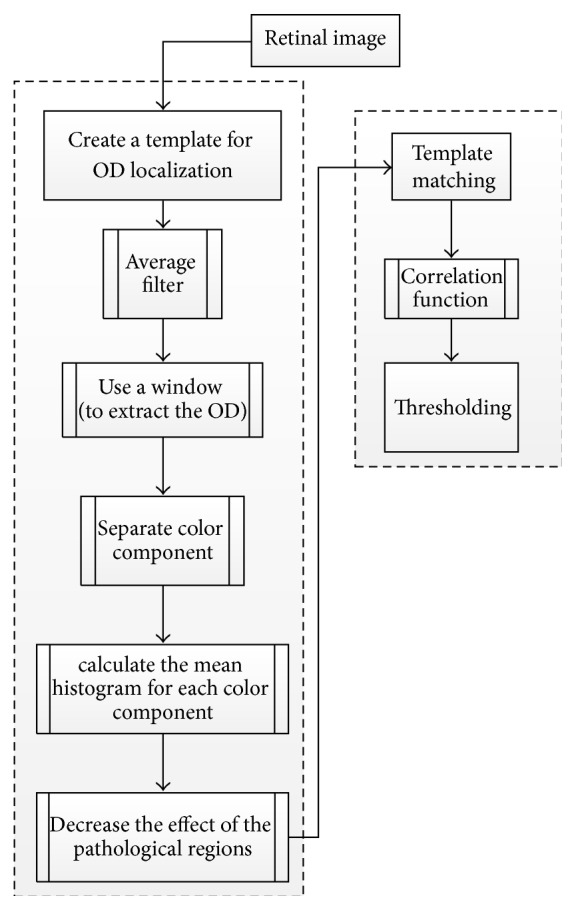
Flowchart for algorithm proposed in [37].

Dehghani and colleagues [37] proposed a novel technique that uses histogram matching for localizing the OD and its center in the presence of pathological regions. The methodology is summarized in Figure 18. Four retinal images from DRIVE dataset were used to create three histograms from the color image components (red, blue, and green) as a template. An average filter was applied to the image to reduce noise. The next step included extracting the OD for each retinal image using a window with a typical size of the OD. Then a template was created by obtaining a histogram for each color component for each OD and calculating the mean of the aforementioned histograms. To reduce the effect of pathological regions with high intensity, the histograms with intensity of lower than 200 were used. The correlation between the histograms of each channel was calculated in order to gain the similarity of two histograms. Finally, thresholding was applied on the correlation function to localize the center of the OD. The methodology was applied on three datasets: 40 images from DRIVE, 273 images from a local dataset, and 81 images from STARE. The success rates were 100%, 98.9%, and 91.36% for the datasets, respectively. In the future work, the OD center will be used as the first step for localizing the boundary as well as for human recognition based on the retinal image.

**Figure 19:**
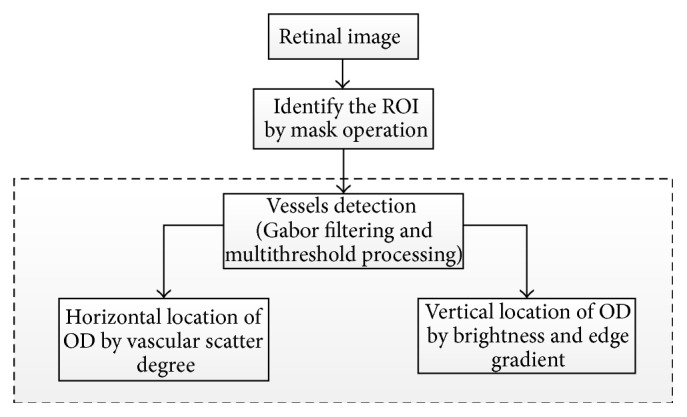
Flowchart for algorithm proposed in [38].

Zhang et al. [38] proposed a novel OD localization technique based on 1D projection (Figure 19). The vascular scatter degree was used to determine the horizontal location of OD. The vertical location of OD was obtained by brightness and edge gradient around OD. A preprocessing step was necessary in which a binary mask obtained by morphological erosion operation was used to identify the region of interest of the retinal image. Blood vessels extraction was then conducted using nonvessel boundary suppression based on Gabor filtering and multithresholding process [53]. The structure of the main vessels is more critical in measurement of vascular scatter degree; therefore, vessels smaller than 30 pixels were neglected. After preprocessing, a vertical window was defined and was slid over the vessels map to calculate the vascular scatter degree in order to obtain 1D horizontal projection signal and locate the horizontal location of the OD at the minimum position of horizontal projection curve. Then a rectangular window was defined, centered at horizontal location of OD, and slid over Gabor filter map and gray intensity image to obtain the 1D vertical projection signal, where the location of the maximum peak of vertical projection curve was the vertical location of the OD. The algorithm was evaluated on four publicly available and one self-marked dataset: (1) 40 images from DRIVE (achieved 100% success rate); (2) 81 images from STARE (achieved 91.4% success rate); (3) 130 images from DIARETDB0 (achieved 95.5% success rate); (4) 89 images from DIARETDB1 (achieved 92.1% success rate); and (5) 40 images from self-selection (achieved 97.5% success rate). Future studies should test the algorithm using a larger dataset.

**Figure 20:**
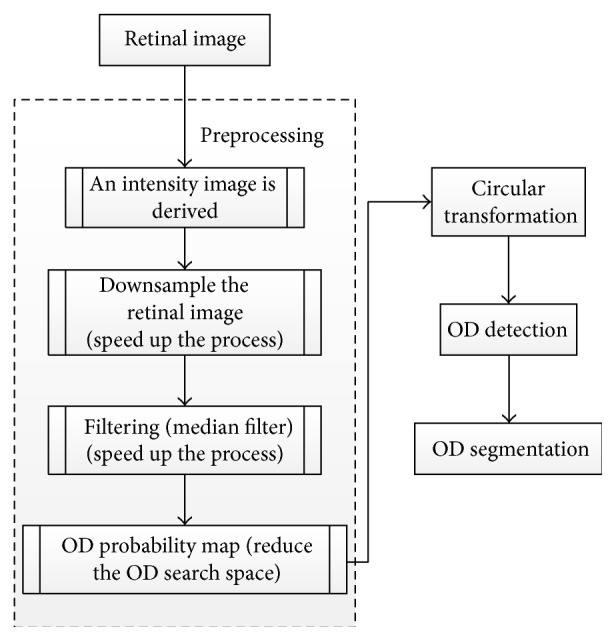
Flowchart for algorithm proposed in [36].

Lu [36] proposed an alternative technique for automatic segmentation of OD (Figure 20). The technique is based on a circular transformation other than Hough. The circular transformation was conducted to detect the circular boundary and color variation across the OD boundary simultaneously. A preprocessing step was essential to improve the accuracy of OD segmentation. The intensity image was first derived from the given retinal image by combining the red and green components since these components contain most of the structural information about OD. Several operations were performed to speed up the process and to improve the accuracy. To decrease the computation cost, image size was reduced to one-third. Then the image was filtered by a median filter to repress speckle noise as well as variation across the retinal vessels. The OD search space was minimized using the OD probability map based on Mahfouz's method [54]. Designing the circular transformation was based on observing the variation of the distance from the point within a circular area to the boundary area which reaches the minimum when the point lies exactly at the centroid region. In particular, each pixel detects maximum variation pixels (PMs) along several evenly oriented radial line segments of specific length. In the next step the PMs were filtered and finally the OD map was obtained by converting the image. In this map, the maximum value represents the OD center and the PMs detected for the pixels at the identified OD center lie on the OD boundary. The algorithm was evaluated on three public datasets: MESSIDOR containing 1200 image, ARIA containing 59 images from individuals with diabetes and 61 normal images, and STARE containing 31 normal and 50 pathological images. The OD detection accuracies were 98.77%, 97.5%, and 99.75%, respectively. The OD segmentation technique was applied only on STARE and ARIA datasets, and the accuracies were 93.4% and 91.7%, respectively.

**Figure 21:**
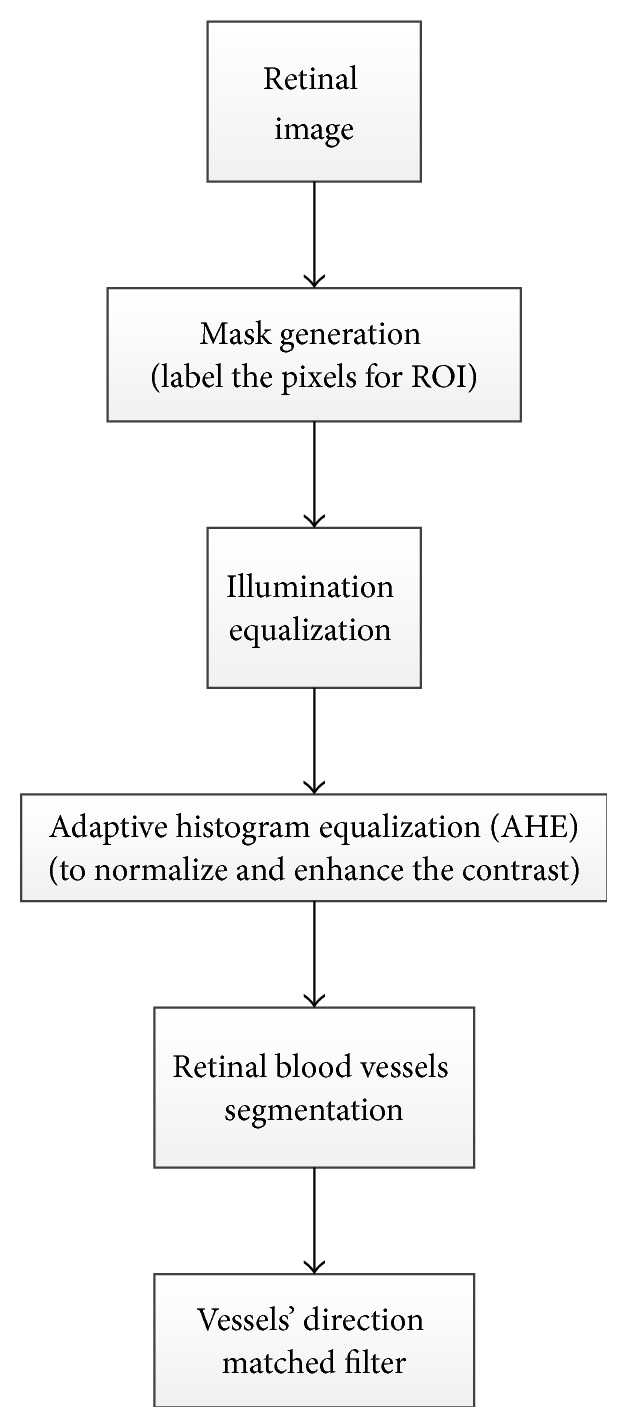
Flowchart for algorithm proposed in [17].

**Figure 22:**
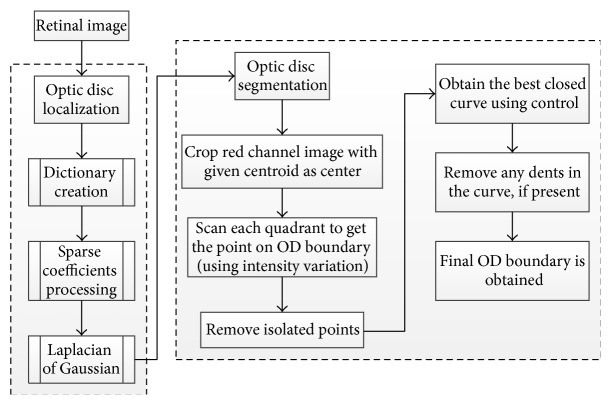
Flowchart for algorithms proposed in [28, 40].

Another OD detection algorithm based on matched filter inspired by the means of vessel direction was introduced by Youssif et al. [17] and is summarized in Figure 21. In the preprocessing step a binary mask was generated by thresholding the red component image, and then a morphological operator was applied to label the pixels on the ROI. The aforementioned was followed by equalizing the illumination using the Hoover and Goldbaum equation [47] to avoid the negative effects of an uneven illumination on OD localization process [56]. The adaptive histogram equalization was applied to improve and normalize the contrast and in turn assist in detecting the small blood vessels with low contrast levels. The blood vessels were segmented based on an algorithm proposed by Chaudhuri et al. [57], where the similarity between the predefined 2D Gaussian template and the fundus image was maximized. To model the retinal vascular in all different orientations, twelve filters were generated to obtain the maximum response for each pixel. To detect the OD, direction match filter was used to match the direction of the vessels at the OD. The algorithm was tested on 40 images from DRIVE dataset and 81 images from STARE dataset, and the success rates were 100% and 98.77%, respectively. The future work should aim to improve blood vessel segmentation by applying other pre- and postprocessing techniques, using other OD parameters or vascular-related OD (e.g., vessel density and diameter), as well as using a larger dataset for testing the algorithm and employing other vessel segmentation algorithms where the vessels direction map can be obtained. A different methodology introduced by Sinha and Babu [40] and Kumar and Sinha [28] is summarized in Figure 22.

The methodology had two main parts. The first part was OD localization using L1 minimization [40] in which a scale embedded dictionary was created based on manually marked fixed-size subimages with OD at the center. These subimages were represented as a column vector to obtain the dictionary elements. Two sets of sparse coefficients, one for the gray intensity image and the other for the red channel image, were obtained. The information from sparse coefficient of each subimage was converted to a single value termed confidence measure. Confidence measure calculated the probability of the OD center falling in a given subimage. The dot products of the confidence values were obtained. The dot products were rearranged over the 2D image grid to form the probability map representing the possibility of finding the OD. A convolution operation was conducted with Laplacian of Gaussian (LoG) blob detector on the map and the location with the most response was declared as the OD. The second part of this methodology was OD segmentation [28]. The method considered the differences between the intensity of OD region and the surrounding area. To simplify the process, the search space was minimized by cropping the red channel. The maximum intensity variation points along both horizontal and vertical directions were obtained. The points that did not lie on the OD boundary were considered “false” points and were removed. A Bezier curve was defined by a set of control points to obtain the best closed curve.

**Figure 23:**
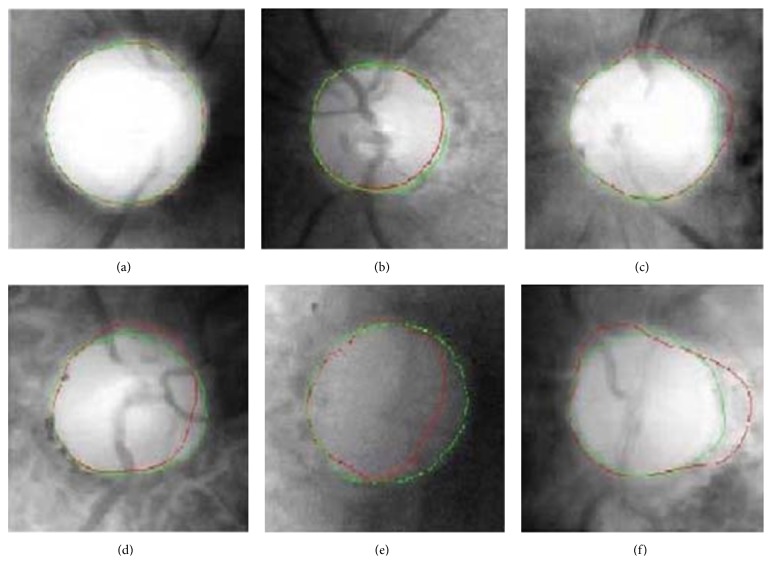
Representative results.

The curve was then smoothened to obtain the final OD boundary (Figure 23).

The localization algorithm [40] was evaluated on multiple datasets DIARETDB0, DIARETDB1, and DRIVE and proved to be successful in 253 out of the total of 259 images from the three dataset (97.68% success rate). The segmentation algorithm [28] was evaluated on 152 images based on two dataset: DIARETDB1 and MESSIDOR. The average overlapping obtained was 89.5%.

### 3.2. Optic Cup Segmentation Approaches

Due to the high density of blood vessels in the optic cup, segmentation in this region is more difficult than optic disc segmentation. Furthermore, the gradual intensity change between the cup and neuroretinal rim causes extra complications for cup segmentation. In addition, glaucoma changes the shape of the cup region. The OD and OC segmentation techniques, in addition to the techniques used only for OC segmentation, are illustrated in Table 4 and the performance results shown in Table 5.

**Figure 24:**
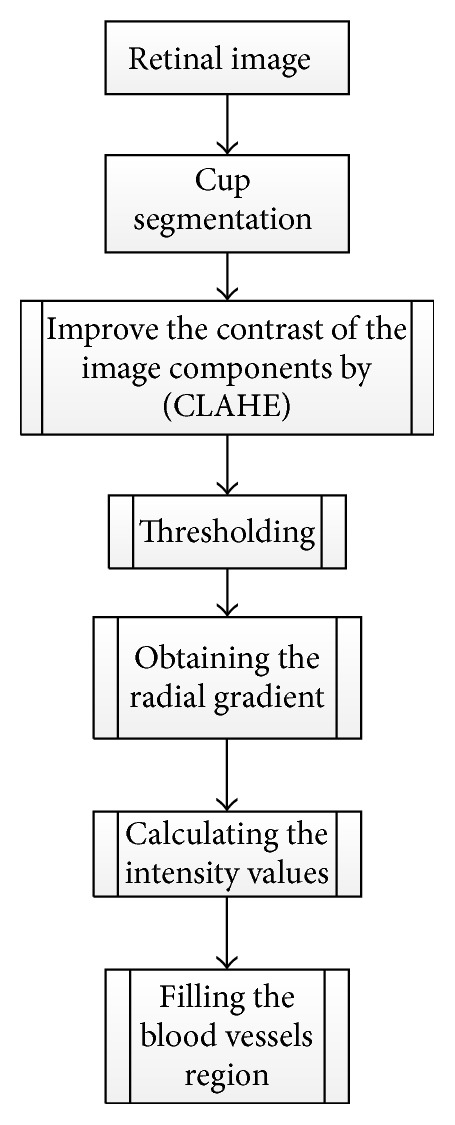
Flowchart for algorithm proposed in [55].

Ingle and Mishra [55] discuss the cup segmentation based on gradient method (Figure 24).

Gradient is the variation in the intensity or color of an image. The gradient images were obtained from an original image convolved with a filter. Two methods were used to find the gradient: (1) linear gradient, (2) radial gradient. The contrast was improved for all image components (red, blue, and green) by Contrast Limited Adaptive Histogram Equalization [67].

The initial threshold was set for red (R), blue (B), and G (green) components after much iteration to detect the region where R channel pixel value is less than 60 and B and G pixel values are greater than 100. Subsequently, other pixels were eliminated by shifting their values to zero. Then the radial gradient was obtained in the images in all directions. The intensities were computed and linearly transformed to the range of (0-1). The G and B channels were considered more effective for OC segmentation. The circular structural elements were used to fill the blood vessels region in order to obtain a continuous region. The algorithm was evaluated based on the accuracy of the cup and disc area in all directions as well as CDR, instead of relying on the accuracy only in one direction. The algorithm can be extended to distinguish between the glaucomatous and normal images.

**Figure 25:**
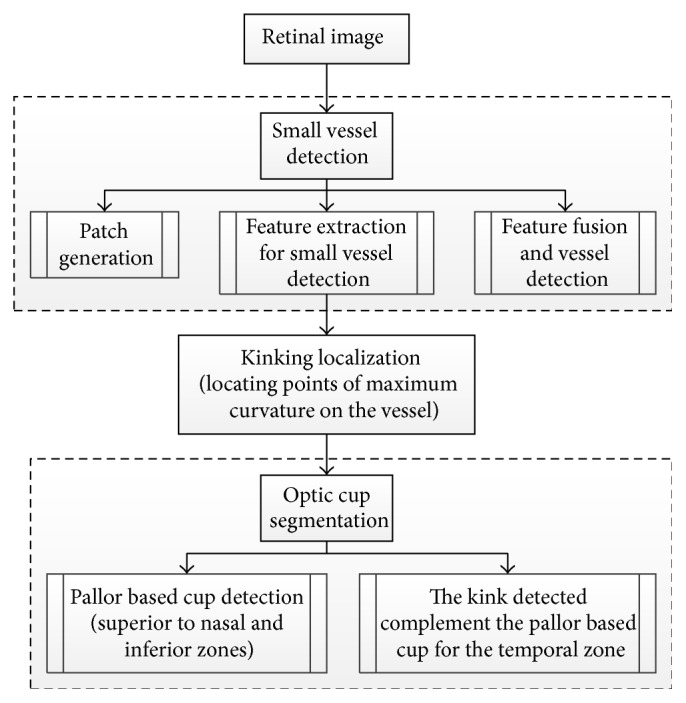
Flowchart for algorithm proposed in [66].

Another system for automatic detection of the optic cup proposed by Damon et al. [66] is based on vessel kinking (Figure 20). To detect the kinks, first the vessels must be detected. The smaller vessels are harder to detect. Therefore, a segmentation technique for small vessel detection was introduced by fusing pixel features and a support vector based classification. Patches of interest (POI) were generated within the optic nerve head. Then features for detecting small vessels were generated, where the green channel was chosen for the feature generation due to its better visibility for the vessels. A wavelet transform was generated for each POI using Gabor filter to detect the overall architecture of vessels. A Canny edge detector was applied to detect all possible vessels. Finally, the feature in the vessels segment based approach was fused instead of pixel classification for the vessels and nonvessels. Kinking was localized by analyzing the identified vessels segments and locating points of maximum curvature on the vessels (i.e., to fit the segment to a curve). To avoid over or under fitting, a rigorous, nonparametric method was used based on the multiscale shifting window technique. Consequently, the optic cup contour was recognized. The pallor-based cup detection was conducted to detect the cup from the superior to nasal and inferior zones. However, the temporal zone was detected by the kinks. The algorithm was tested on 67 images from the SERI.  Figure 25 shows flowchart for algorithm proposed in [66].

### 3.3. Optic Disc and Optic Cup Segmentation

To calculate the CDR and ISNT, the optic disc and optic cup should be segmented simultaneously. The increased intraocular pressure in glaucoma increases the cup size. Therefore, changes in the cup size and CDR are considered important indications of glaucoma [6, 68]. The neuroretinal rim is an effective factor in glaucoma evaluation according to the ISNT rule, when the optic disc and cup are precisely detected [45, 69].

**Figure 26:**
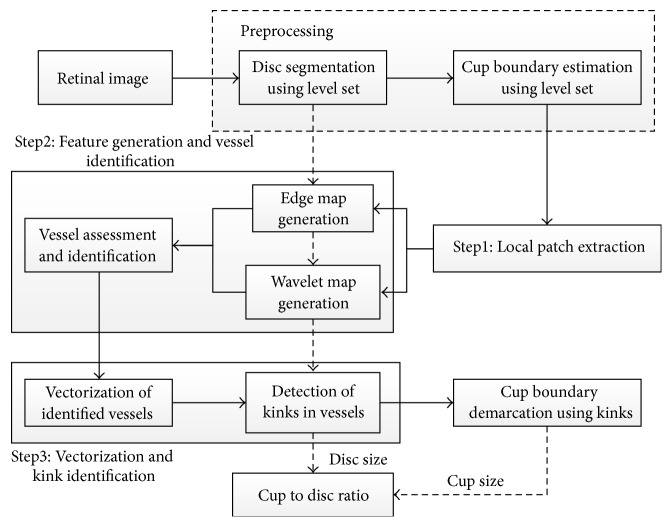
Flowchart for algorithm proposed in [59].

Wong et al. [59] described a novel technique for detecting blood vessel kinks for optic cup segmentation (Figure 26). A preprocessing step was conducted using a level set method to obtain the optic disc and to estimate the initial optic cup boundary. Therefore, a region of interest was extracted and the disc center was identified by thresholding of the red component. Next, variational level set method [70] and direct ellipse fitting approach [71] were applied to obtain and smoothen the optic disc. The initial contour was obtained by extracting the OD region in green channel. The results were then ellipse-fitted in order to provide an approximation of the cup boundary based on pallor. Square pixel size patches were used as guide based on the pallor cup boundary to locate the kinks within the optic disc. Canny edge detection and wavelet transform were applied separately in the green channel to detect the kinks on the intradisc vessel edges. To avoid the effects of protrusion along some of the detected edges, a polynomial application was used to smooth each edge, followed by vectorizing the vessel edges and dividing them into 15 segments. The kinks were identified by calculating the change in the angle between each of the two edges. Finally, the kinks and the additional points with the direct ellipse fitting method were used to determine the OC boundary. The algorithm was evaluated with 27 images from SERI.

The CDR calculated by the kink and pallor methods were compared with the ground truth CDR and the average errors of each method was calculated. The average errors were 0.139 and 0.093 for pallor method and kink method, respectively.

**Figure 27:**
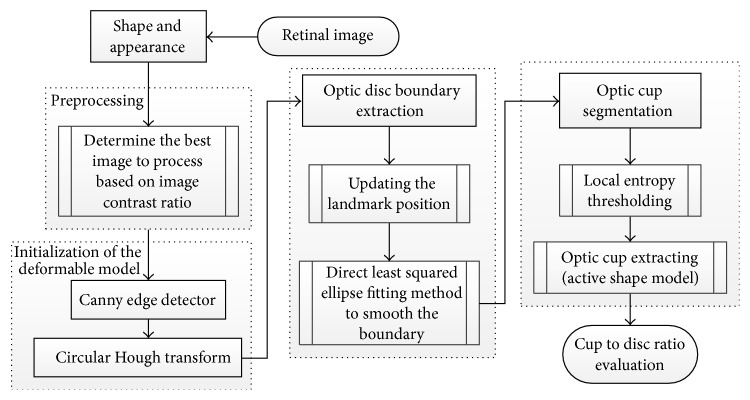
Flowchart for algorithm proposed in [63].

Yin et al. [63] introduced a statistical model based method that combines circular Hough transform and a novel optimal channel selection for OD and OC segmentation. The method is summarized in Figure 27.

**Figure 28:**
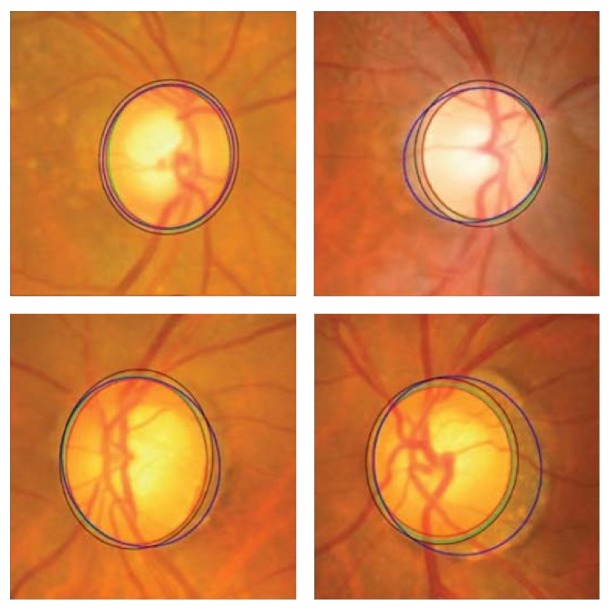
OD segmentation using proposed method (red), level set method (blue), and FCM method (black) with ground truth (green).

**Figure 29:**
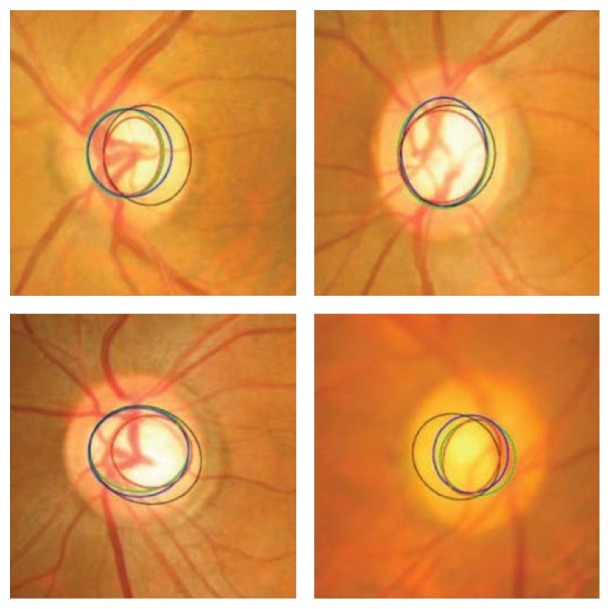
Optic cup segmentation using the proposed method (blue), ASM method without vessel removal (red), and level set method (black) with ground truth (green).

The active shape model using 24 landmark points around the OD was used as the first step. A preprocessing was conducted to decrease the effect of blood vessels, and the best image was determined based on the image contrast ratio. Identifying the OD center and approximating the OD size requires a good initialization. Therefore, Canny edge detector and circular Hough transform were applied to obtain the edge map and approximate the OD, respectively. Then the statistical deformable model was initialized to adjust the OD boundary. To update the OD segmentation, the landmark position by minimizing the Mahalanobis distance was conducted, followed by the direct least squared ellipse fitting method to smooth the boundary of the contour (Figure 28). On the other hand, the OC boundary was extracted by applying the active shape model in the green channel of the image without blood vessel. The optic cup center is close to the OD center; therefore, the model was initialized by translating the mean cup model to the OD center (Figure 29). The ORIGA dataset consisting of 650 images was used to evaluate the algorithm. The average Dice coefficient for the OD and OC segmentation was 0.92 and 0.81, respectively. The mean absolute CDR error was 0.10.

**Figure 30:**
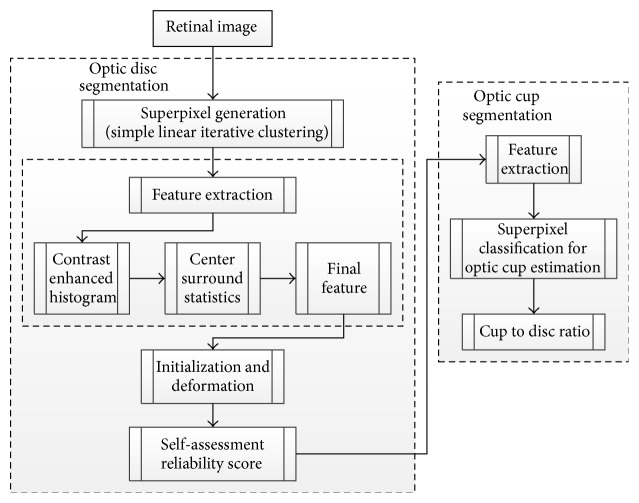
Flowchart for algorithm proposed in [6].

Superpixel classification based optic disc and optic cup segmentation for glaucoma screening system was introduced by Cheng et al. [6] and is illustrated in Figure 30. Classifying each superpixel as disc or nondisc in the OD segmentation was done based on histograms and center surround statistics. On the other hand, in the OC segmentation the location information was also included.

**Figure 31:**
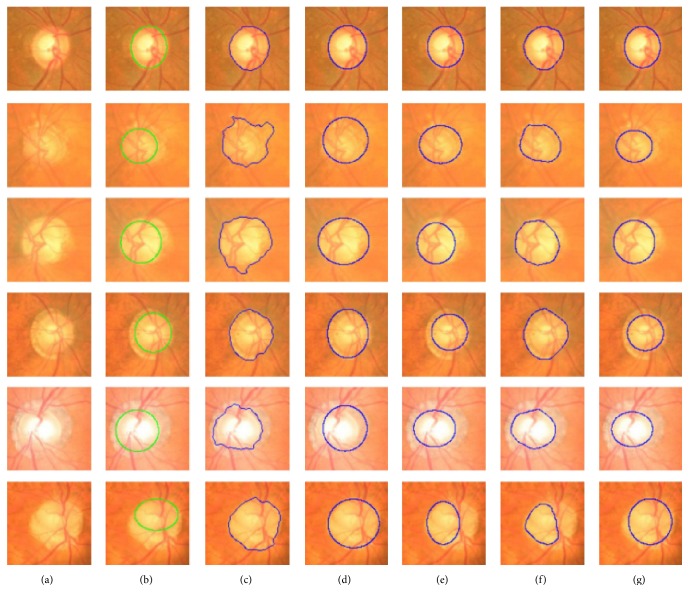
Sample results of the optic disc. From left to right columns: (a) the original images, (b) the manual “ground truth,” and ((c)–(g)) outlines by the MCV, CHT-ASM, EHT, and MDM.

**Figure 32:**
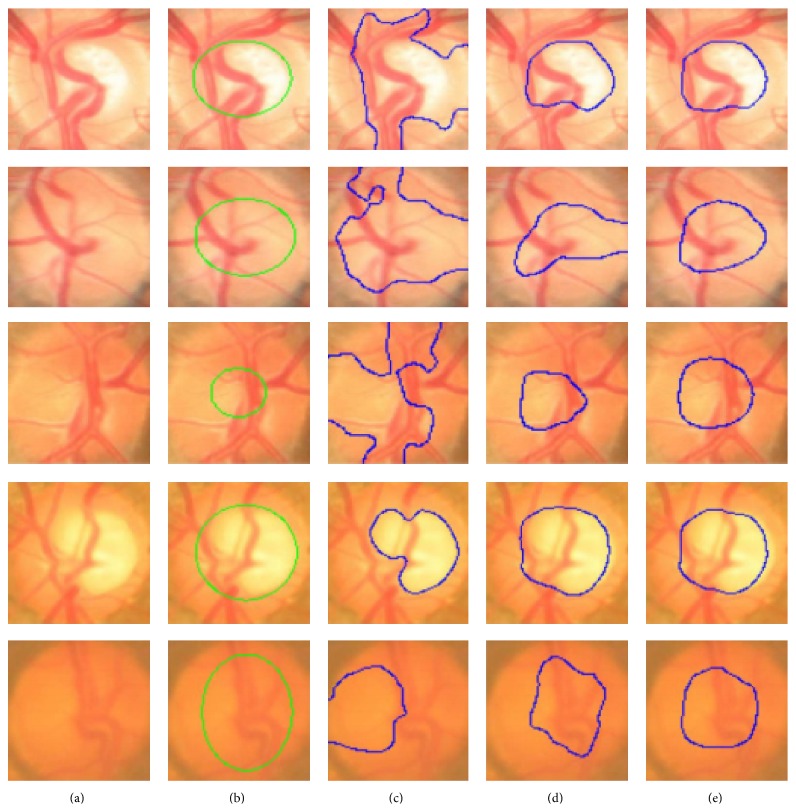
Sample results of the optic cup. From left to right columns: (a) the original images, (b) the manual “ground truth,” and ((c)–(e)) outlines by the proposed method before ellipse fitting.

A Simple Linear Iterative Clustering algorithm [72] was used to gather nearby pixels into superpixels. Extracting OD features was achieved by enhancing the contrast using the histogram equalization for the three image components (R, B, and G) and computing the center surround statistics to avoid color similarity in the group of pixels forming the superpixel. A Library for Support Vector Machine (LIBSVM) [73] was used as classifier to extract the OD boundary (Figure 31). Detecting the OC boundary was based on the feature extraction where the histogram feature was computed. Red channel histograms were excluded. The center surround statistics was computed similar to the OC feature extraction. Finally, LIBSVM was used as classifier to extract the OC boundary (Figure 32). Knowing the OD and OC, the CDR could be computed. The algorithm was evaluated based on 2326 images from two resources: SiMES and SCEN. Results showed an average overlapping error of 9.5% in optic disc segmentation and 24.1% in optic cup segmentation using only the SiMES dataset.

**Figure 33:**
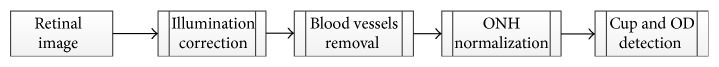
Flowchart for algorithm proposed in [62].

Mishra et al. [62] proposed an active contour method to find the CDR in order to determine glaucoma (Figure 33). The green channel image was used in the segmentation process similar to the previous algorithms. Illumination was corrected using a mathematical morphology in which the background of the image was estimated by morphological opening process. The blood vessels were removed by applying a morphology based vessel segmentation proposed by Fraz and colleagues [74]. Subsequently, image inpainting was used to replace the blood vessel region with plausible background. Multithresholding and active contour method were used to determine the OD and OC boundaries. Thus, the CDR could be calculated. The method was tested on 25 images obtained from an optic disc organization in UK. Preprocessing techniques were required to improve the results.

**Figure 34:**
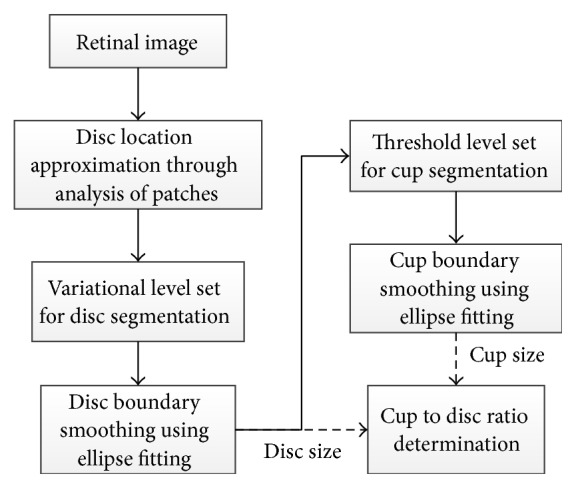
Flowchart for algorithm proposed in [58].

Wong et al. [58] proposed an automatic CDR detection algorithm based on a variational level set approach (Figure 34). Localizing the OD using intensity information was the first step. Therefore, an image histogram was obtained in which pixels with the highest intensity were selected to be the disc region. Also, the image was divided into 64 regions, and the disc center was the region with greatest number of high intensity pixels. The ROI was identified by a circle with a radius twice as long as the typical normal OD radius. The variational level set algorithm was applied to detect the OD boundary using red channel. Next, ellipse fitting was applied to smooth the boundary. Due to the high density of the blood vessels in the OC region, the green channel was selected to be processed. The OC was segmented by applying threshold initialized level set from the segmented disc. The boundary was smoothed by ellipse fitting. The CDR was calculated as the final result. The methodology was evaluated using 104 images from SiMES and the results produced up to 0.2 CDR units from manually graded samples.

**Figure 35:**
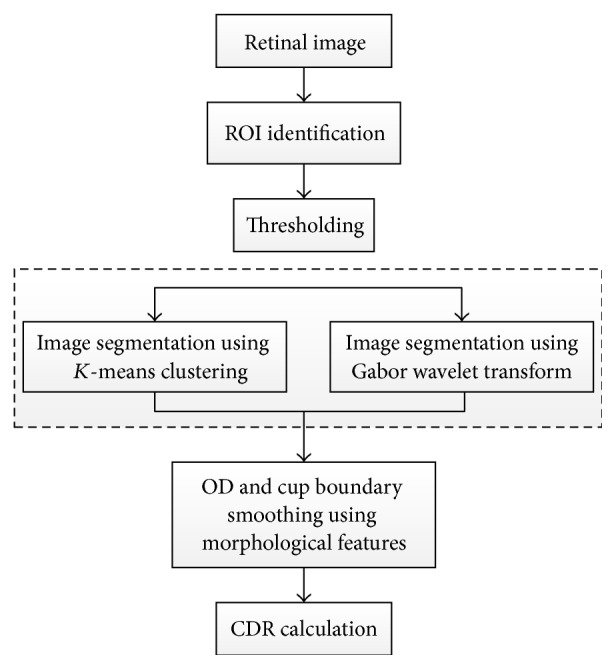
Flowchart for algorithm proposed in [65].

To calculate CDR, *K*-mean pixel clustering technique and Gabor wavelet transform [65] were used to segment the OD and OC separately (Figure 35). The *K*-mean clustering classifies the data into a number of clusters. For each cluster *K* centroids were defined and each point in the data was associated to the nearest centroid to create groups. The first step is completed when no point is pending and an early group is created. Then *K* was recalculated to new centroids as barycenters of the clusters of the previous step. As the result, a new binding had to be made between the same data and the nearest new centroid. A loop was created to track the location of the centroids until centroids did not move any more in order to reduce the objective function (squared error function). The *K*-mean clustering was conducted on ROI identified by a mask. Finally, OC and OD were segmented using green plane to choose mean value for background blood vessel. Then the disc and cup were replaced, where the image was mapped in 4 iterations to calculate the mean value of the matrix distance.

Morphological feature was performed to smooth the cup and disc boundary. Gabor wavelet transform was also executed to avoid problems due to the presence of blood vessels. Since the vessels have directional pattern, the Gabor wavelet transform was tuned for specific frequencies and orientations to filter out the background noise.

**Figure 36:**
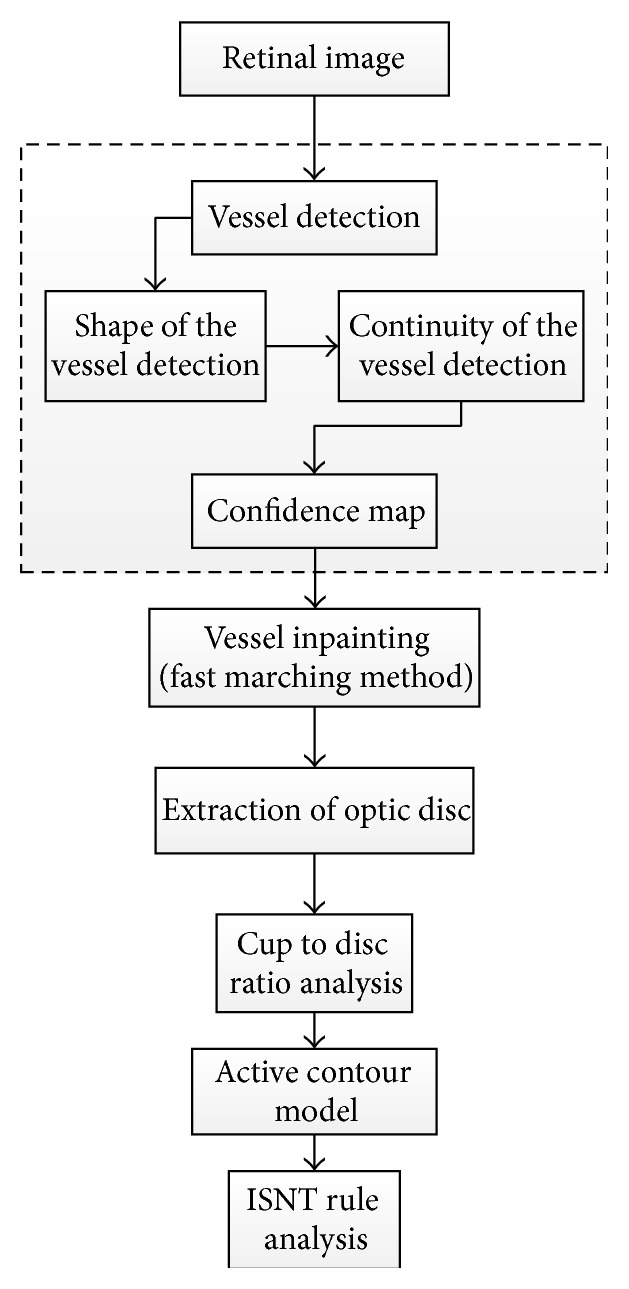
Flowchart for algorithm proposed in [61].

Ho et al. [61] developed a novel technique for automatic fundus image analysis for glaucoma screening (Figure 36). The technique involved two major steps. Detecting the blood vessels was the initial step and was conducted using two structural characteristics: shape and continuity feature. A Canny edge detector was applied to detect general edges containing the boundaries of blood vessels, where the green channel was used in the analysis. Bayesian rules were used to generate an accurate confidence map by combining the horizontal and vertical confidence maps from the shape and continuity feature. Fast Marching Method was employed to fill the vessels free spaces and then the peak thresholding from inpainted image histogram was executed for segmentation, where the image was segmented into three regions. Firstly, in order to estimate the disc boundary, the three regions were fitted with two circles and the active contour model was applied to extract the boundaries of the inner cup and surrounding disc. Then the CDR parameter and ISNT rule were calculated. A dynamic histogram equalization technique may be applied to enhance the image contrast and to avoid wrong identification of the CDR and ISNT due to nonclear OC on vessels free images.

**Figure 37:**
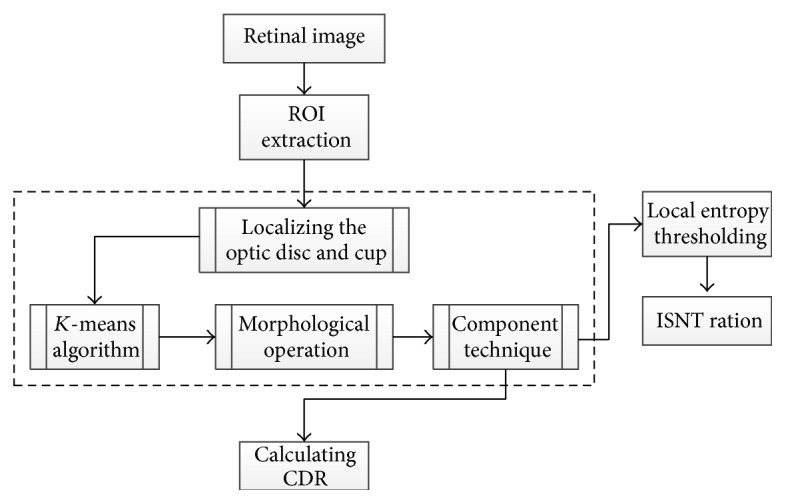
Flowchart for algorithm proposed in [60].

Narasimhan and Vijayarekha [60] introduced a new system for glaucoma detection based on *K*-mean clustering technique to extract OD and OC and also an elliptical fitting technique to calculate the CDR. In addition, a local entropy thresholding approach was applied to detect the blood vessels and compute ISNT. The system consists of three phases (Figure 37). The first phase was ROI extraction considering green plane. The second phase was the feature extraction through *K*-mean clustering [65]. ROI covered the OD, OC, and small part of the region near to the OD in the retinal image; thus, the *K* value was chosen as 3. The clusters not belonging to the OD and OC were removed. As the result, two clusters from the OD region remained, since the operation was conducted to fill the holes and spaces inside OD and OC clusters.

Subsequently connected component technique was applied to form rectangles that represented the entire OD and OC. To calculate CDR, elliptical fitting technique was executed on OD and OC and the areas of the ellipse, OC, and OC were computed using a specific formula. The ISNT was computed by measuring the area of the blood vessels in the four quadrants. Therefore, a local entropy thresholding was used to segment the blood vessels and then a mask was applied to measure the four areas. Three classifiers, that is, KNN, BAYES, and SVM, were used to test 15 normal and 21 glaucomatous images. KNN achieved 93.3% and 80.9% success rates; BAYES achieved 86.6% and 95.23% success rates; and SVM achieved 100% 95.23% success rates for normal and abnormal images, respectively.

**Figure 38:**
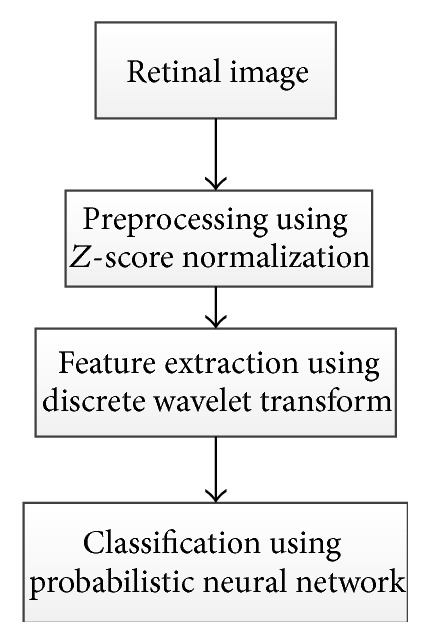
Flowchart for algorithm proposed in [42].

Annu and Justin [42] proposed another method for automated classification of glaucoma by wavelet energy feature. The technique uses texture features within the image by applying energy distribution over wavelet subbands and efficient glaucoma classification based on Probabilistic Neural Network.  Figure 38 illustrates a summary of the algorithm. The wavelet features were gained from the Daubechies (db3), symlets, and biorthogonal wavelet filters.* Z*-score normalization was applied to the images to equalize the irregular illumination associated with the image. The feature of the retinal image was extracted to simplify the classification process since it provides characteristics of input pixel to the classifier. Therefore, the wavelet transform was applied. Various textures have different energy in the space frequency domain; hence, the energy obtained from the coefficient was used to distinguish between the normal and glaucomatous images. Finally, a Probabilistic Neural Network was used as classifier to analyze image properties and classify the dataset. This involved two phases: a training phase and a testing phase. In the training phase the known data was given and in the testing phase the unknown data was used. The algorithm was applied on 10 normal and 10 glaucomatous images, and 15 images were used for training. The results showed sensitivity, specificity, positive predictive accuracy, and accuracy of 100%, 90%, 90%, and 95%, respectively.

**Figure 39:**
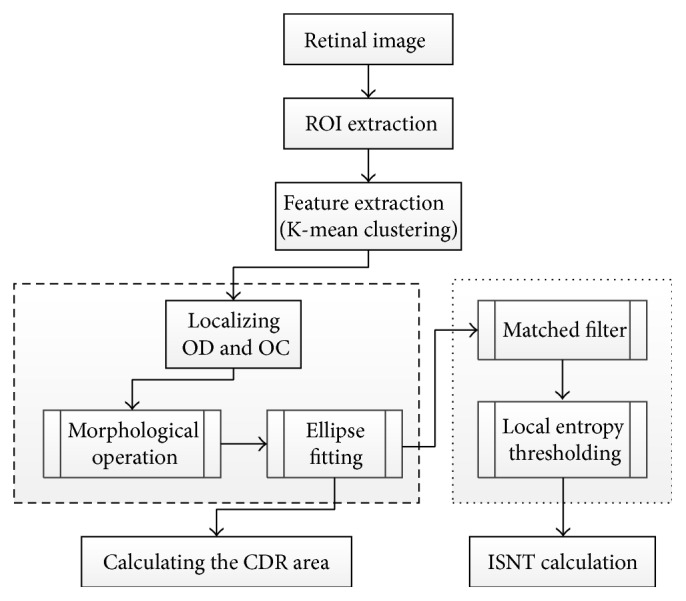
Flowchart for algorithm proposed in [64].

The OD and OC segmentation technique proposed by Narasimhan and colleagues [64] implements the openCV library functions based on *K*-mean clustering and elliptic fitting (Figure 39) to calculate CDR.

The cvMinAreaRect2() was used to draw the ellipse and the blood vessels were extracted using matched filter in which cv2DRotationMatrix and cvWarpAffine were used to rotate the kernel. Finally local entropy thresholding was applied to compute ISNT ratio. The openCV was mainly used to increase the operation speed. The method was evaluated on 50 images obtained from Aravind Eye Hospital in India.

## 4. Discussion

We provided a comprehensive review of the algorithms used for OD and OC detection and segmentation that help with diagnosis of glaucoma by detecting the main structures of the ONH. Many algorithms were limited due to the complexities of ONH structure which is very variable among people and among different pathologies. The variabilities in the ONH structure also cause difficulties in diagnostic observations. Papillary atrophy causes some difficulties for disc segmentation due to its similarity in intensity to disc boundaries. However, there are some algorithms that can segment the disc with PPA perfectly. On the other hand, disc drusen causes greater difficulty for segmenting the disc since the changes that appear on the disc boundaries make the discs completely covered, especially in advanced cases. No current segmentation technique considers disc drusen based on retinal fundus images due to the rarity of the case and its complexity in terms of image processing. Blood vessels play an important role in accurate segmentation of cup boundaries. This is a challenge facing many researchers and there are limited segmentation techniques that address this challenge. The algorithms performed differently depending on the datasets of images. Some approaches used a small dataset, while some used large datasets to train and test the algorithm. Many methods were tested only on normal retinal images, and those that were evaluated on pathological images used different number of glaucomatous images. Also, the severity of the disease was different among the datasets used in different techniques; therefore, the corresponding algorithms cannot be compared with each other.

Most of the OD segmentation was based on the circular Hough transform along with other detection techniques. Aquino et al. [32] obtained excellent results based on a large dataset (MESSIDOR) for both localization and segmentation of OD; however, errors occurred due to ellipse eccentricity that was not suitable for circular approach. Yin et al. [34] introduced an edge detection, circular Hough transform to estimate the OD center and diameter, and a statistical deformable model to adjust the disc boundary according to the image texture. Utilizing a large dataset, the method gained good results. Cheng et al. [35] also achieved good results by eliminating the peripapillary atrophy based on edge filtering, constraint elliptical Hough transform, and peripapillary atrophy detection. Due to the low contrast of the disc boundary, OD boundary was not detected in some images. Therefore, a preprocessing stage to select the best image component is necessary to improve the results. Lu [36] gained excellent results based on three datasets: STARE, ARIA, and MESSIDOR. A circular transform was designed to determine the circular shape of OD and also the image variation across the OD boundary with a very short computation time of only 5s. Three sources of error contributed to segmentation failure for a few images; these were as follows: (1) the large color variation across the OC boundary might have caused the PMs to fall on the cup boundary instead of disc boundary, (2) due to ultralow image variation across the OD boundary, much of the OD boundary had no PM detected and therefore the OD boundary remained undetected, and (3) the error introduced by the PMs was created based on symmetry. On the other hand, Cheng et al. [6] obtained perfect results using an OD and OC segmentation algorithm based on superpixel classification utilizing histograms and center surround statistics. However, a preprocessing step was essential to improve the image. Also, to create a robust algorithm multiple kernel learning [75] was required for enhancement and extraction of blood vessels to fine-tune the cup boundary. Sinha and Babu [40] proposed an algorithm to localize the OD based on L1 minimization. The algorithm achieved a high success rate in localizing the OD; the algorithm failed in only 6 out of 259 images. The fails were due to the scaling factor which needs to be fixed at the start of a trial for the dataset to downsample the image to suitable size in tune with those in the dictionary. The OD detection was then followed by segmenting technique [28]. This approach sometimes results in false segmentation due to the blood vessels and nerves crossing the OD that appear darker than OD and therefore restrict the search space in the retinal image.

In general, in addition to blood vessel extraction, a preprocessing step including image channel selection, illumination normalization, and contrast improvement is necessary for a robust approach in OD and OC segmentation. Retinal pathological images that have captured the effect of the disease on the optic nerve head must be considered in order to obtain correct computations of the CDR and ISNT for the glaucoma screening. Precise OD and OC localization lead to perfect segmentation. The aforementioned highlights the importance of utilizing an accurate localization technique. Evaluating algorithm based on various datasets will increase the reliability of the outcomes.

## 5. Conclusion

Segmentation of the optic disc and optic cup has captured the interest of many researchers. Although there are many promising approaches, there is still room for improvement in segmentation techniques. Only few of the existing methodologies, whether for optic disc or for optic cup segmentation, can be applied for glaucomatous retinal images. Also, most of the current methods have been tested on a limited number of datasets such as DRIVE and STARE. These datasets do not provide images with many different characteristics. Furthermore, the generally low resolution of the images (ranging from 0.4 to 0.3 megapixels) has made the segmentation process even more challenging [76]. An advanced camera capable of taking high volumes of high resolution retinal images will facilitate glaucoma screening. In order to achieve good outcomes for the images captured by different systems, robust and fast segmentation methods are required. Most of the retinal images used to evaluate segmentation methods have been taken from adults. The retinas of infants, babies, and children have different morphological characteristics than that of adults, and this difference must be considered in segmentation methodologies [76]. The glaucoma screening system complements but does not replace the work of ophthalmologists and optometrists in diagnosis; routine examinations have to be conducted in addition to the fundus image analysis. However, the system facilitates diagnosis by calculating the disc and cup structural parameters and showing greater details of ONH, such as the disc and cup areas, the vertical and horizontal cup-to-disc ratios, and cup to disc area ratio, and also checking the ISNT arrangement. This is a shareable opinion that could associate the worlds of consultant ophthalmologists, optometrists, orthoptists, and engineers.

The main contribution of this paper is in introducing a survey of current optic disc and optic cup segmentation methods for calculating the CDR and ISNT. The optic disc segmentation methods were covered first, followed by two optic cup segmentation methods. Finally, the optic disc with optic cup segmentation methods were covered. The main objective was to present some of the current detection and segmentation methodologies and to give the reader an overview of the existing research. The current trends and challenges and the future directions in optic disc and optic cup segmentation were also discussed.

## Figures and Tables

**Table 1:** Performance metrics for optic disc and optic cup segmentation.

Measurement	Description
Optic disc overlap	TP/TP + FN + FP
CDR error	CDR (GR) − CDR (PRP)
ISNT rule	Optic disc-optic cup (obtain the thickness in all the four quadrants)
Specificity (SP)	TN/TN + FP
Sensitivity (SN)	TP/TP + FN
Accuracy (Acc)	TP + TN/TP + FP + FN + TN
Positive predictive accuracy (PPA)	TP/TP + FP
Dice metric (DM)	2 *∗* TP/FP + TP + FN
Relative area difference (RAD)	FP + FN/GT

**Table 2:** Optic disc segmentation methods.

Authors	Year	Image processing technique	Performance metrics	Dataset	Number of images
Lupaşcu et al. [29]	2008	Circles passing through three noncollinear points	Success rate	DRIVE	40

Youssif et al. [17]	2008	Normalized DFI by means of a vessels' direction matched filter	Success rate	STARE	81

Zhu and Rangayyan [30]	2008	Edge detection using canny and sobel methods and through transform	Success rate	DRIVE STARE	40 82

Welfer et al. [31]	2010	Adaptive morphological approach	Overlap (Acc)	DRIVE DIARETDB1	40 89

Aquino et al. [32]	2010	Morphological, edge detecting, and feature extraction techniques	Overlap (Acc)	MESSIDOR	1200

Tjandrasa et al. [33]	2012	Hough transform and active contours	Overlap (Acc)	DRIVE	30

Yin et al. [34]	2011	Model based segmentation	Overlap (Acc)	ORIGA	650

Cheng et al. [35]	2011	Peripapillary atrophy elimination	Overlapping error	ORIGA	650

Lu [36]	2011	Circular transformation	Overlap (Acc)	MESSIDOR ARIA STARE	1200 120 81

Dehghani et al. [37]	2012	Histogram matching	Success rate	DRIVE STARE Local	40 81 237

Zhang et al. [38]	2012	Projection with vessel distribution and appearance characteristics	Success rate	DRIVE	40

Fraga et al. [39]	2012	Fuzzy convergence and hough transform	Success rate	VARIA	120

Sinha and Babu [40]	2012	Optic disc localization using L1 minimization	Overlap (Acc)	DIARETDB0 DIARETDB1 DRIVE	130 89 40

Kumar and Sinha [28]	2013	Maximum intensity variation	Overlap (Acc) SN	MESSIDOR DIARETDB0	40 130

**Table 3:** Performance results for the optic disc segmentation.

Authors	Year	Database	Sensitivity	Average overlapping	Overlap error	Success rates (Acc)	Computation time (s)
Lupaşcu et al. [29]	2008	DRIVE				95% localization 70% identification of OD	60

Youssif et al. [17]	2008	DRIVE STARE				100% localization 98.77% localization	210

Zhu and Rangayyan [30]	2008	DRIVE STARE				92.5% 40.24%	N/A

Welfer et al. [31]	2010	DRIVE DIARETDB1				100% 97.7%	1083

Aquino et al. [32]	2010	MESSIDOR				99% localization 86% segmentation	1.67 5.69

Yin et al. [34]	2011	ORIGA			11.3%		N/A

Cheng et al. [35]	2011	ORIGA			10%		N/A

Lu [36]	2011	MESSIDOR ARIA STARE				98.77% detection 97.5% detection, 91.7% segmentation 99.75% detection, 93.4% segmentation	5

Tjandrasa et al. [33]	2012	DRIVE				75.56%	N/A

Fraga et al. [39]	2012	VARIA				100% localization 93.36% segmentation	0.6

Dehghani et al. [37]	2012	DRIVE STARE Local				100% 91% 98.9%	27.6

Zhang et al. [38]	2012	DRIVE Self-selection STARE DIARETDB0 DIARETDB1				100% 97.5% 91.4% 95.5% 92.1%	13.2

Sinha and Babu [40]	2012	DIARETDB0 DIARETDB1 DRIVE				96.9% 100% 95%	3.8

Kumar and Sinha [28]	2013	MESSIDOR DIARETDB0	93%	0.895			90

**Table 4:** Categorization optic disc with optic cup segmentation methods.

Authors	Year	Image processing technique	OD/OC	Performance metrics	Datasets	Number of images
Wong et al. [58]	2008	Variational level-set approach	OD/OC	CDR	SERI	104

Wong et al. [59]	2009	Vessel kinking	OD/OC	CDR	SERI	27

Narasimhan and Vijayarekha [60]	2011	*K*-mean clustering	OD/OC	CDR-ISNT ratio	AEH	36

Ho et al. [61]	2011	Inpainting and active contour model	OD/OC	CDR-ISNT ratio	CMUH	N/A

Mishra et al. [62]	2011	Active contour method	OD/OC	CDR	ODO, UK	25

Yin et al. [63]	2012	Model-based segmentation	OD/OC	DM, RAD	ORIGA	650

Narasimhan et al. [64]	2012	*K*-means and openCV code	OD/OC	CDR-ISNT ratio	AEH	50

Cheng et al. [6]	2013	Superpixel classification	OD/OC	CDR	ORIGA + SCES	2326

Annu and Justin [42]	2013	Wavelet energy features	OD/OC	SN – SP – Acc – PPA	N/A	20

Chandrika and Nirmala [65]	2013	*K*-means clustering and Gabor wavelet transform	OD/OC	CDR	N/A	N/A

Damon et al. [66]	2012	Vessel kinking	OC	Overlap error	SERI	67

Ingle and Mishra [55]	2013	Gradient method	OC	N/A	N/A	N/A

**Table 5:** Performance results for the optic disc and optic cup segmentation.

Authors	Year	Database	OD/OC	Sensitivity	Specificity	Overlap error	Success rate (Acc)	AUC	Computation time (s)
Wong et al. [58]	2008	ORIGA	OD/OC			4.81%			NA

Wong et al. [59]	2009	SERI	OD/OC	0.813	0.455				NA

Narasimhan and Vijayarekha [60]	2011	AEH	OD/OC				95%		NA

Ho et al. [61]	2011	CMUH	OD/OC	N/A	NA	NA	NA		NA

Mishra et al. [62]	2011	ODO	OD/OC				100%		NA

Yin et al. [63]	2012	ORIGA	OD/OC			9.72% (OD) 32% (OC)			NA

Narasimhan et al. [64]	2012	AEH	OD/OC	NA	NA	NA	NA	NA	NA

Cheng et al. [6]	2013	ORIGA + SCES	OD/OC			9.5% (OD) 24.1% (OC)		0.800 (ORIGA) 0.822 (SCES)	10.9 (OD) 2.6 (OC)

Annu and Justin [42]	2013	NA	OD/OC	100%	90%		95%		NA

Chandrika and Nirmala [65]	2013	NA	OD/OC	NA	NA	NA	NA	NA	NA

Damon et al. [66]	2012	SERI	OC			N/A			NA

Ingle and Mishra [55]	2013	NA	OC	NA	NA	NA	NA	NA	NA
